# Understanding host-microbiota interactions in the commercial piglet around weaning

**DOI:** 10.1038/s41598-021-02754-6

**Published:** 2021-12-06

**Authors:** M. Saladrigas-García, M. D’Angelo, H. L. Ko, P. Nolis, Y. Ramayo-Caldas, J. M. Folch, P. Llonch, D. Solà-Oriol, J. F. Pérez, S. M. Martín-Orúe

**Affiliations:** 1 Animal Nutrition and Welfare Service (SNiBA), Department of Animal and Food Science, Universitat Autònoma de Barcelona (UAB), 08193 Bellaterra, Spain; 2 Nuclear Magnetic Resonance (SeRMN), Universitat Autònoma de Barcelona (UAB), 08193 Bellaterra, Spain; 3 Animal Breeding and Genetics Program, Institute for Research and Technology in Food and Agriculture (IRTA), Torre Marimon, 08140 Caldes de Montbui, Spain; 4 Plant and Animal Genomics, Centre for Research in Agricultural Genomics (CRAG), CSIC-IRTA-UAB-UB Consortium, 08193 Bellaterra, Spain; 5 Department of Animal and Food Science, Universitat Autònoma de Barcelona (UAB), 08193 Bellaterra, Spain

**Keywords:** Gene expression, Microbial communities, Next-generation sequencing, Metabolomics, Gastrointestinal system, Genomics

## Abstract

Weaning is a critical period in the life of pigs with repercussions on their health and welfare and on the economy of the swine industry. This study aimed to assess the effect of the commercial early weaning on gut microbiota, intestinal gene expression and serum metabolomic response via an integrated-omic approach combining 16S rRNA gene sequencing, the OpenArray gene expression technology and ^1^H-NMR spectroscopy. Fourteen piglets from different litters were sampled for blood, jejunum tissue and caecal content two days before (− 2d), and three days after (+ 3d) weaning. A clearly differential ordination of caecal microbiota was observed. Higher abundances of *Roseburia*, *Ruminococcus*, *Coprococcus*, *Dorea* and *Lachnospira* genera in weaned piglets compared to prior to weaning showed the quick microbial changes of the piglets’ gut microbiota. Downregulation of *OCLN*, *CLDN4*, *MUC2*, *MUC13*, *SLC15A1* and *SLC13A1* genes, also evidenced the negative impact of weaning on gut barrier and digestive functions. Metabolomic approach pinpointed significant decreases in choline, LDL, triglycerides, fatty acids, alanine and isoleucine and increases in 3-hydroxybutyrate after weaning. Moreover, the correlation between microbiota and metabolome datasets revealed the existence of metabolic clusters interrelated to different bacterial clusters. Our results demonstrate the impact of weaning stress on the piglet and give insights regarding the associations between gut microbiota and the animal gene activity and metabolic response.

## Introduction

The process of weaning is one of the most stressful events for pigs in swine production. Pigs are subjected to biological stress marked by significant physiological, social, environmental and nutritional challenges. Weaning is usually performed at around 3–4 weeks, when piglets are still vulnerable to infectious diseases because of stress and immaturity of intestinal tract and immune system^1^. Therefore, during the first week of adaptation to solid feed, piglets experience low voluntary feed intake, which results in alteration of gut integrity^2,3^, characterized by shortened villous length^4^ and increased mucosal permeability^5,6^. As a result, a reduced pig health and performance is observed in commercial practice. In order to improve pig health and welfare during this time, a better understanding of the complex phenomena underlying is needed.

Recently, the association among gut microbiota, metabolites and host physiology has gained increasing attention. The animal organism is believed to be interconnected with its environment through different cycles of epigenomic programming and reprogramming^7^. This epigenomic programming is the result of the interaction of metabolism and the microbiota, as well as their interaction with external factors such as diet, environmental exposure or drugs. On the one hand, the process of intestinal microbial colonization has been shown to play a crucial role in the development of the neonatal immune system of mammals with implications throughout their lives^8^. Early gut microbial colonization is affected by factors such as age, host genetics, diet, environment, disease and maternal seeding^9^. This, in turn, sets off the crosstalk between the microbiome and the host driving changes in nutrition, immunity, barrier function, metabolism, and gene expression. Actually, gut microbiota has been described to be involved in the intestinal epithelium differentiation^10^, the immune system development^11^, and intestinal mucosal barrier maintenance^12^. An adequate gut colonization in the piglet is therefore considered as a key element to maintain the homeostasis and promote an optimal training of the immune response with lifelong implications on the probability of developing pathologies^13^, such as post-weaning diarrhoea or other multifactorial diseases. On the other hand, it is well known that the intestinal microbiota plays a key role in gut health, participating in many metabolic pathways, such as the nutrient digestion, absorption or lipid metabolism, and amino acid synthesis^14^. However, few studies have focused on the complex interactions that occur in the pig organism during the suckling and weaning period.

The objective of this work was therefore to provide further clarity in the complex crosstalk between the piglets and their intestinal microbiota early in life. For that, we studied changes in the gut ecosystem and the animal response under different commercial husbandry practices. We assessed changes on gut microbiota, intestinal gene expression and serum metabolic profiles in piglets via an integrated omics approach combining 16S rRNA gene V3-V4 regions sequencing, the TaqMan OpenArray gene expression technology and Proton Nuclear Magnetic Resonance (^1^H-NMR) spectroscopy.

## Results

This work was part of a larger study that has been previously published^15–17^ that is recommended for complementary information. In those publications we evaluated the impact of different management systems (including or not early socialization and environmental enrichment) on behavioural response^15^, performance^16^, and intestinal physiology and caecal microbiota^17^ of piglets. In the present work, our objective was to elucidate the changes induced by the commercial early weaning itself attending to changes on caecal microbiota, intestinal gene expression and metabolomic response. We also aimed to integrate all these data in a holistic approach to better understand the relationships between microbiota and animal response.

### Weaning-induced changes in gut bacterial microbiome

**Figure 1:**
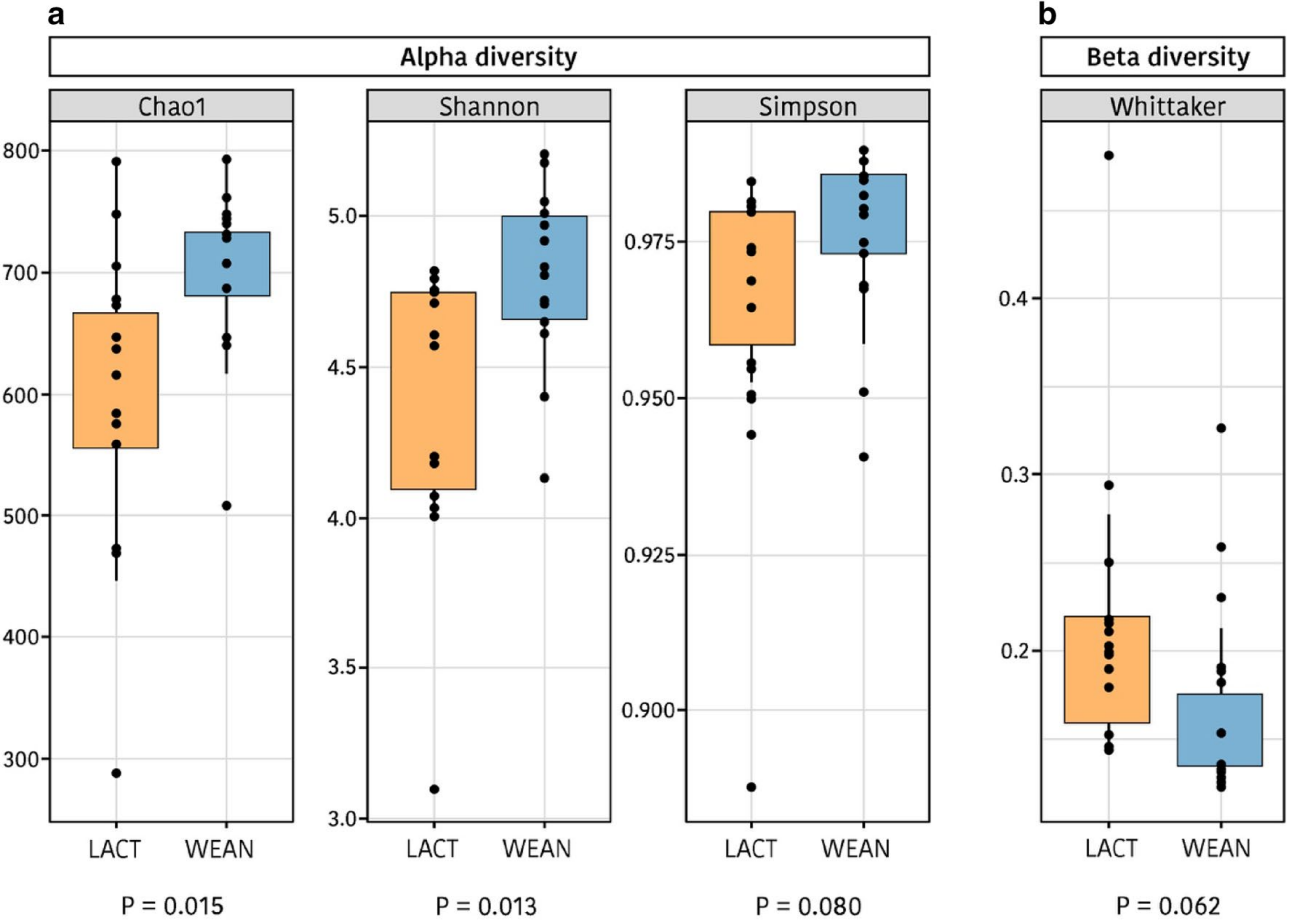
Box plot of the alpha (**a**) and beta (**b**) diversity during lactation (LACT) and after weaning (WEAN) based on the calculation of different indices: Chao1, Shannon and Simpson for alpha diversity, and Whittaker for beta-diversity.

An average of 78,562 ± 24,539 16S rRNA gene V3-V4 regions sequences per sample (ranging from 40,061 to 132,201) with an average length of 460 bp were obtained from the 28 caecal content samples, with no differences in the number of reads between pre- and post-weaning piglets (*P* = 0.424) and rarefaction curves reaching the plateau phase. The sequences were assigned to 976 Operational Taxonomic Units (OTU) based on a 97% sequence similarity. The indices of *Chao1*, *observed species*, *Shannon* and *Simpson* were calculated to estimate alpha diversity. As presented in Fig. 1., higher values were found after weaning (*P* = 0.015, *P* = 0.017, *P* = 0.013, *P* = 0.080; for *Chao1*, *observed species*, *Shannon* and *Simpson* indices, respectively), indicating an increase in the complexity of the gut ecosystem when animals are moved to the dry feed. Regarding beta diversity, a tendency was detected with the Whittaker index for a decrease after weaning (*P* = 0.062) indicating that ecosystems were more similar and a microbiota trend to converge between animals after weaning. Moreover, analysis of possible changes in the ecosystem structure related to weaning were performed using Anosim, Adonis and Envfit tests, all of them based on Bray–Curtis distance. Highly significant differences between pre- and post-weaning piglets were found (*P* = 0.0001, *P* = 0.001 and *P* = 0.0001, for Envfit, Anosim and Adonis tests, respectively).

### Changes promoted in particular taxonomic groups

**Figure 2:**
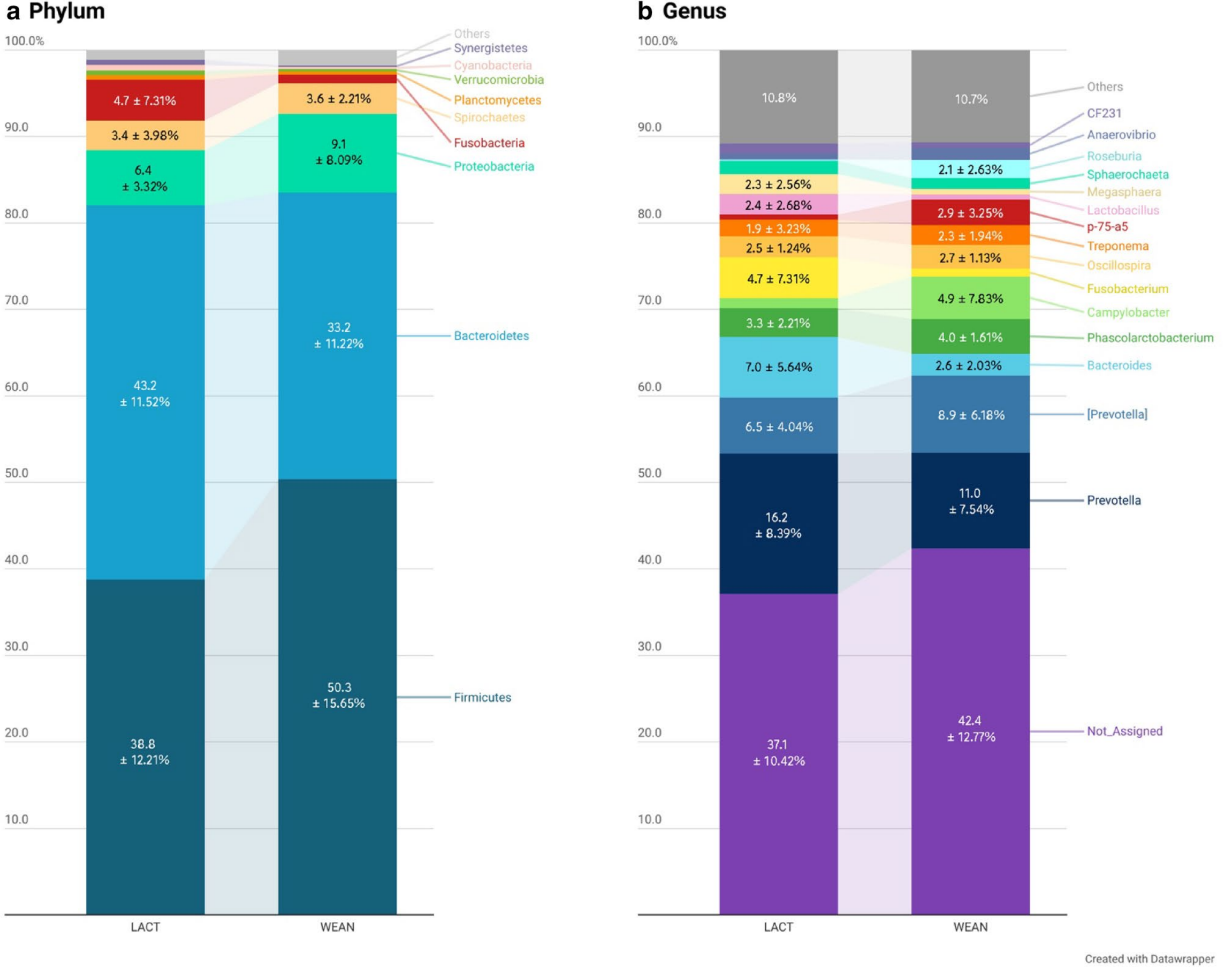
Bar plot of the relative abundances (RA) expressed in percentage of the phyla (**a**) and main genera (**b**) observed in the analysis of the microbiota of piglets by massive sequencing of the 16S rRNA gene. Bar plot LACT represents the relative abundances observed during lactation, while bar plot WEAN represents the values observed in weaned piglets. Only taxa with RA greater than 2.0% were annotated with RA percentage ± SD. Figure created with the online open-source tool Datawrapper (http://datawrapper.de).

Figure 2 shows the relative abundances obtained for suckling and weaned piglets at phylum and genus levels. *Firmicutes* and *Bacteroidetes* constituted the two predominant phyla in the caecal microbiota of the piglets, followed by *Proteobacteria* (7.76%), *Spirochaetes* (3.49%) and *Fusobacteria* (2.85%). At genus level, *Prevotella* was found as the most predominant genus (13.61%), followed by unclassified *Prevotella* (7.70%) and *Bacteroides* (4.79%) from *Bacteroidetes* phyla.

**Figure 3:**
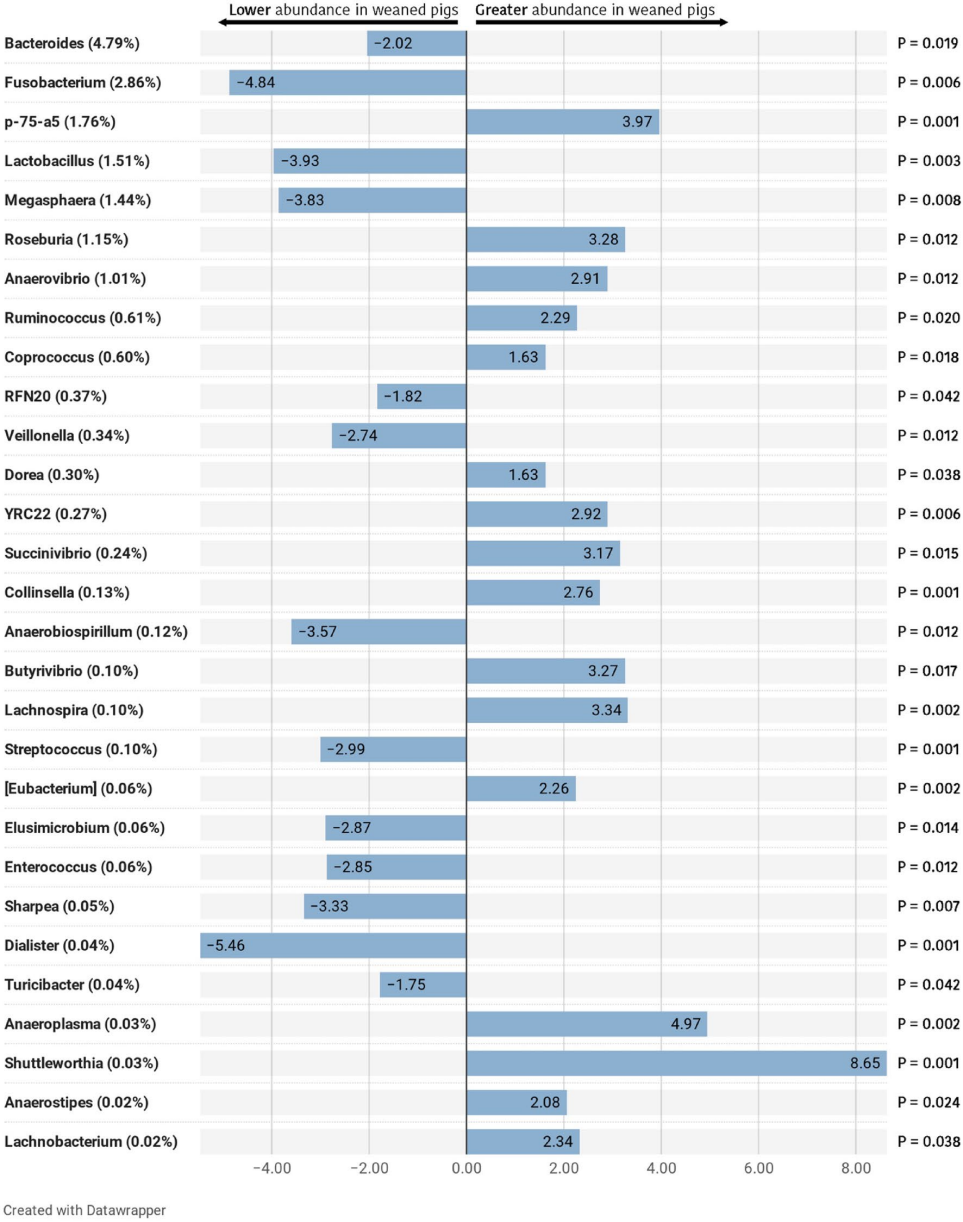
Differentially abundant taxa from caecal content (ln change and adjusted *P*-value < 0.05) between suckling and weaned piglets at genus level. All significant genera are presented; positive values and negative values indicate greater and lower abundance, respectively, in weaned animals (WEAN group) compared to suckling piglets (LACT); taxa are sorted according to the general mean of relative abundance (the average of LACT and WEAN, indicated between brackets in %) and in decreasing order. Figure created with the online open-source tool Datawrapper (http://datawrapper.de).

Regarding the effect of weaning on microbial groups, although the most abundant phyla, *Firmicutes*, *Bacteroidetes* and *Proteobacteria* revealed no significant shifts in their relative abundances as a whole, significant effects were seen in predominant family and genera within. Only *Fusobacteria* phylum showed a remarkable decrease after weaning (*P* = 0.005). At the family level, four predominant families showed significant reductions as the piglets were weaned (Table 1), including *Bacteroidaceae* (*P* = 0.031), *Enterobacteriaceae* (*P* = 0.031), *Fusobacteriaceae* (*P* = 0.005), and *Lactobacillaceae* (*P* = 0.003). At the same time, *Lachnospiraceae* and *Erysipelotrichaceae* increased significantly after weaning (*P* = 0.031 and *P* = 0.022, respectively). At the genus level, two predominant genera showed significant increases after weaning (Fig. 3), including *p-75-a5* (*P* = 0.001) and *Roseburia* (*P* = 0.012). The relative abundances of *Bacteroides*, *Fusobacterium*, *Lactobacillus*, and *Megasphaera* decreased in weaned piglets. Notably, *Bacteroides* and *Fusobacterium*, which were abundant in the gut of suckling piglets, declined from 7.01 and 4.72% to 2.58 and 0.99%, respectively for *Bacteroides* (*P* = 0.019) and *Fusobacterium* (*P* = 0.006), in a 5-day period. Among other non-predominant genera, several significant shifts were also detected, as for example, an increased abundance of *Ruminococcus*, *Coprococcus*, *Dorea* and *Lachnospira* in weaned piglets (*P* = 0.020; *P* = 0.018; *P* = 0.038; and *P* = 0.002, respectively).

**Table 1:** Composition of the caecal microbiota of piglets at family level.

	LACT	WEAN	SEM	*P*-value
*Ruminococcaceae*	12.92	18.51	1.404	0.6061
*Prevotellaceae*	16.21	10.94	1.555	0.2545
*[Paraprevotellaceae]*	8.64	10.16	0.993	0.9756
*Veillonellaceae*	7.98	6.67	0.854	0.6361
*Lachnospiraceae*	5.06	9.22	1.276	**0.0311**
*Bacteroidaceae*	7.00	2.58	0.894	**0.0311**
*S24-7*	3.84	3.61	0.342	0.6779
*Campylobacteraceae*	1.18	4.86	1.104	0.2384
*Fusobacteriaceae*	4.72	0.99	1.071	**0.0050**
*Erysipelotrichaceae*	1.45	3.81	0.496	**0.0216**
*Clostridiaceae*	1.80	3.22	0.296	0.1376
*Spirochaetaceae*	1.89	2.30	0.495	0.2317
*Lactobacillaceae*	2.40	0.63	0.422	**0.0033**
*Sphaerochaetaceae*	1.51	1.26	0.306	0.9756
*Desulfovibrionaceae*	0.98	1.15	0.149	0.9756
*Enterobacteriaceae*	1.66	0.44	0.256	**0.0311**
*Pasteurellaceae*	1.28	0.49	0.271	0.0576
*Christensenellaceae*	0.49	1.20	0.184	0.0759
*[Odoribacteraceae]*	1.15	0.48	0.193	0.0747
*Dethiosulfovibrionaceae*	0.48	0.10	0.078	**0.0311**
*Coriobacteriaceae*	0.19	0.39	0.075	**0.0476**
*Victivallaceae*	0.23	0.04	0.046	**0.0042**
*Streptococcaceae*	0.17	0.03	0.021	**0.0025**
*Enterococcaceae*	0.11	0.01	0.022	**0.0216**
*Anaeroplasmataceae*	0.00	0.07	0.033	**0.0025**

Only main families (RA > 1%) and statistically significant families are included. Relative abundance results are expressed as percentage (%) in decreasing order according to the general mean (the average of LACT and WEAN), and with the standard error of the mean (SEM), followed by the adjusted *p*-values (*P*-value) resulting from the comparison between suckling (LACT) and weaned (WEAN) piglets. Significant values are in bold.

### Predicted functions of the caecal microbiota

**Figure 4:**
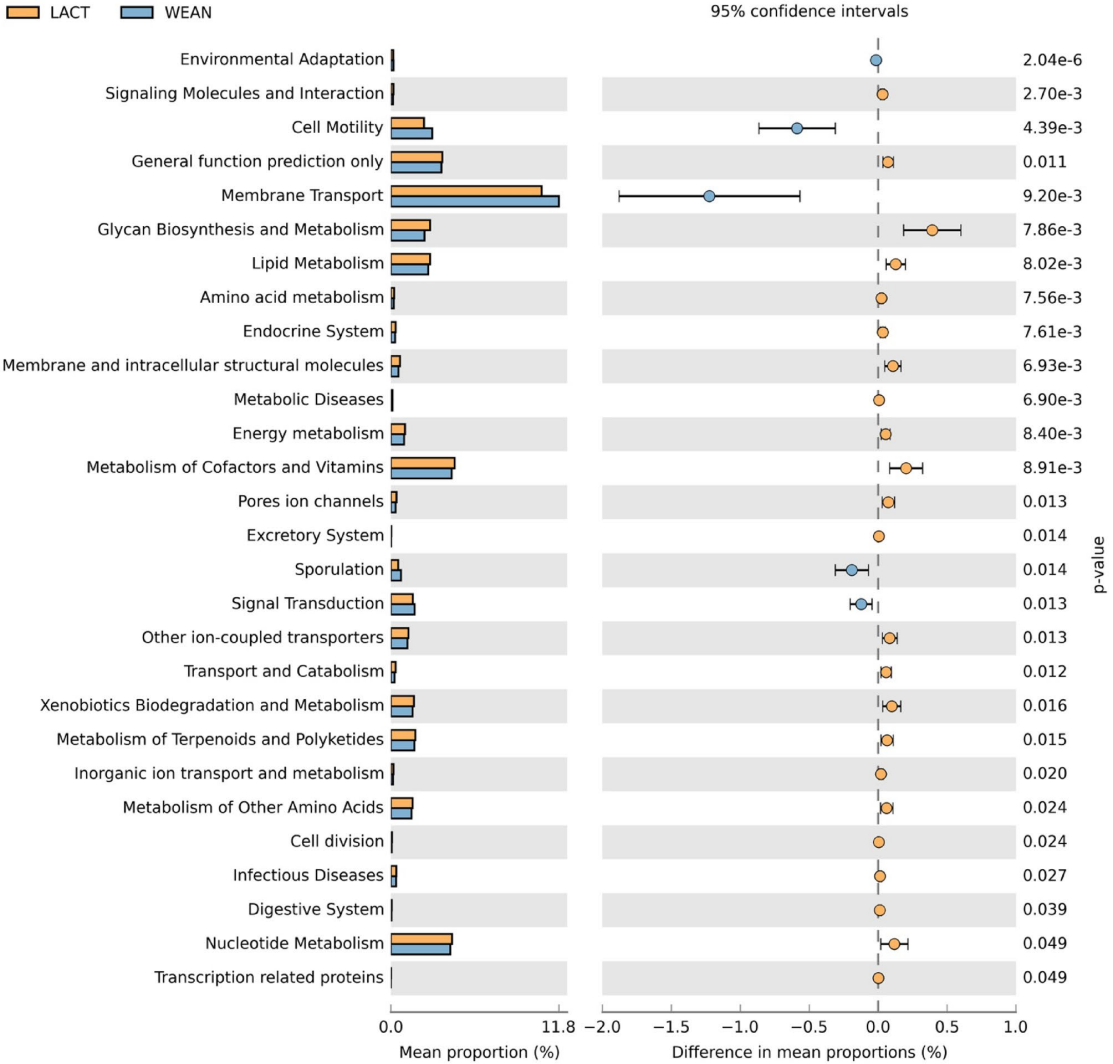
Significant differing caecal microbiota pathways between suckling and weaned piglets (KEGG level 2). All sequence reads were used to predict functions against the KEGG database^19^ (http://www.genome.jp/kegg/) by means of PICRUSt^18^ v.1.1.3. (http://picrust.github.io/picrust/) bioinformatics software package. Difference values are expressed as difference from pre- to post-weaning. Figure created with the software package STAMP^20^ v2.1.3. (https://github.com/dparks1134/STAMP).

The inference of the functional profile of the caecal microbial community was predicted by using PICRUSt^18^ v1.1.3. A clear differentiation was observed between lactating and weaned piglets’ microbiota related to several KEGG (Kyoto Encyclopedia of Genes and Genomes) pathways^19^. During lactation, pathways related to metabolism, like glycan biosynthesis or lipid metabolism, cofactor and vitamin and nucleotide metabolism, were highly represented. After weaning, functions related to cellular processes, as cell motility and sporulation, or related to environment information processing, like membrane transport and signal transduction were higher (Fig. 4).

Going to a deeper level (KEGG level 3, presented in Supplementary Fig. S1), lipid metabolism pathways, such as lipid biosynthesis proteins (*P* = 0.005), energy metabolism pathways, such as carbon fixation pathways (*P* = 0.019) and carbohydrate metabolism pathways, such as the citrate cycle (TCA cycle, *P* = 0.028), among others, were more represented in suckling piglets. The pathways involved in metabolism of purine (*P* = 0.041) and alanine, aspartate and glutamate metabolism (*P* = 0.049), related to nucleotide and amino acid metabolism pathways, respectively, were also higher during lactation compared to suckling. Nicotinate and nicotinamide metabolism (*P* = 0.008), related to the metabolism of cofactors and vitamins, increased also in weaned piglets compared to lactation.

Weaned piglets showed, however, a higher proportion of pathways involved in bacterial chemotaxis (*P* = 0.004), bacterial motility proteins (*P* = 0.007) and flagellar assembly (*P* = 0.014), all related to cell motility. Pathways related with membrane transport, such as transporters (*P* = 0.018) and ABC transporters (*P* = 0.015), and signal transduction, such as the two-component system (*P* = 0.015) were higher after weaning. Finally, lipopolysaccharide biosynthesis proteins (*P* = 0.040), related to the glycan biosynthesis pathway, and sporulation pathway (*P* = 0.014), showed higher values in weaned piglets.

### Changes induced in the jejunal gene expression

Jejunum samples from the fourteen piglets were collected shortly before and after weaning to analyse the expression of several genes related to intestinal functionality by using the OpenArray technology. The 51 genes analysed were grouped into six categories for easier understanding according to whether they were related to: barrier function (BF), immune response (IR), nutrient transport (NT), enzyme/hormone encoders (EH), stress indicators (ST) or housekeeping (HK).

Several genes from all functional groups showed significant changes after weaning as shown in Table 2. Moreover, some additional genes related to barrier function, tended to be downregulated (*CLDN4* and *MUC2,* *P* = 0.064 and *P* = 0.074 respectively) or increased (*CLDN15*, *P* = 0.079) in weaned piglets.

**Table 2:** Statistically significant differences observed in jejunal gene expression between suckling and weaned piglets.

Function	Gene	Suckling (Crt value ± SD)	Weaned (Crt value ± SD)	*P*-value
BF	*OCLN*	7.8 ± 0.47	7.1 ± 0.28	0.0004
BF	*MUC13*	2.4 ± 1.1	1.1 ± 0.35	0.0007
IR	*IFNGR1*	4.7 ± 0.8	3.3 ± 0.35	0.0001
IR	*PPARGC1α*	7.3 ± 0.55	8.0 ± 0.59	0.0154
NT	*SLC16A1*	6.6 ± 0.74	7.8 ± 0.62	0.0014
NT	*SLC15A1*	4.6 ± 1.73	3.5 ± 0.99	0.0176
NT	*SLC13A1*	6.3 ± 2.16	4.4 ± 0.68	0.0098
NT	*SLC30A1*	4.8 ± 0.64	3.7 ± 0.51	0.0003
NT	*SLC39A4*	4.6 ± 0.75	6.1 ± 0.57	0.0002
EH	*SI*	2.9 ± 1.21	1.5 ± 0.74	0.0029
EH	*HNMT*	5.3 ± 0.59	4.6 ± 0.37	0.0031
EH	*CCK*	7.4 ± 0.62	9.5 ± 1.13	0.0002
EH	*IGF1R*	6.3 ± 0.64	7.5 ± 0.74	0.0003
ST	*HSD11B1*	8.8 ± 0.69	9.8 ± 1.01	0.0117

Values are expressed as Crt values ± Standard Deviation. Each analysed gene was classified based on its functionally: barrier function (BF), immune response (IR), nutrient transport (NT), enzyme/hormone encoders (EH) or stress indicators (ST). The *p*-values have been adjusted by the FDR method. A brief description of the genes analysed is given in Supplementary Table S1.

### Weaning-induced changes in serum metabolome

#### ^1^H-NMR spectra

Twenty-eight serum samples were prepared and analysed from 14 suckling piglets and 14 weaned piglets. Among the different endogenous metabolites assigned there were LDL, VLDL, lipids, unsaturated lipids, leucine, valine, isoleucine, lactate, alanine, adipate, acetate, N-acetyl glycoproteins, O-acetyl glycoproteins, glutamine, glutamate, pyruvate, creatine, choline, trimethylamine-N-oxide (TMAO), glucose, creatinine, tyrosine and phenylalanine.

**Figure 5:**
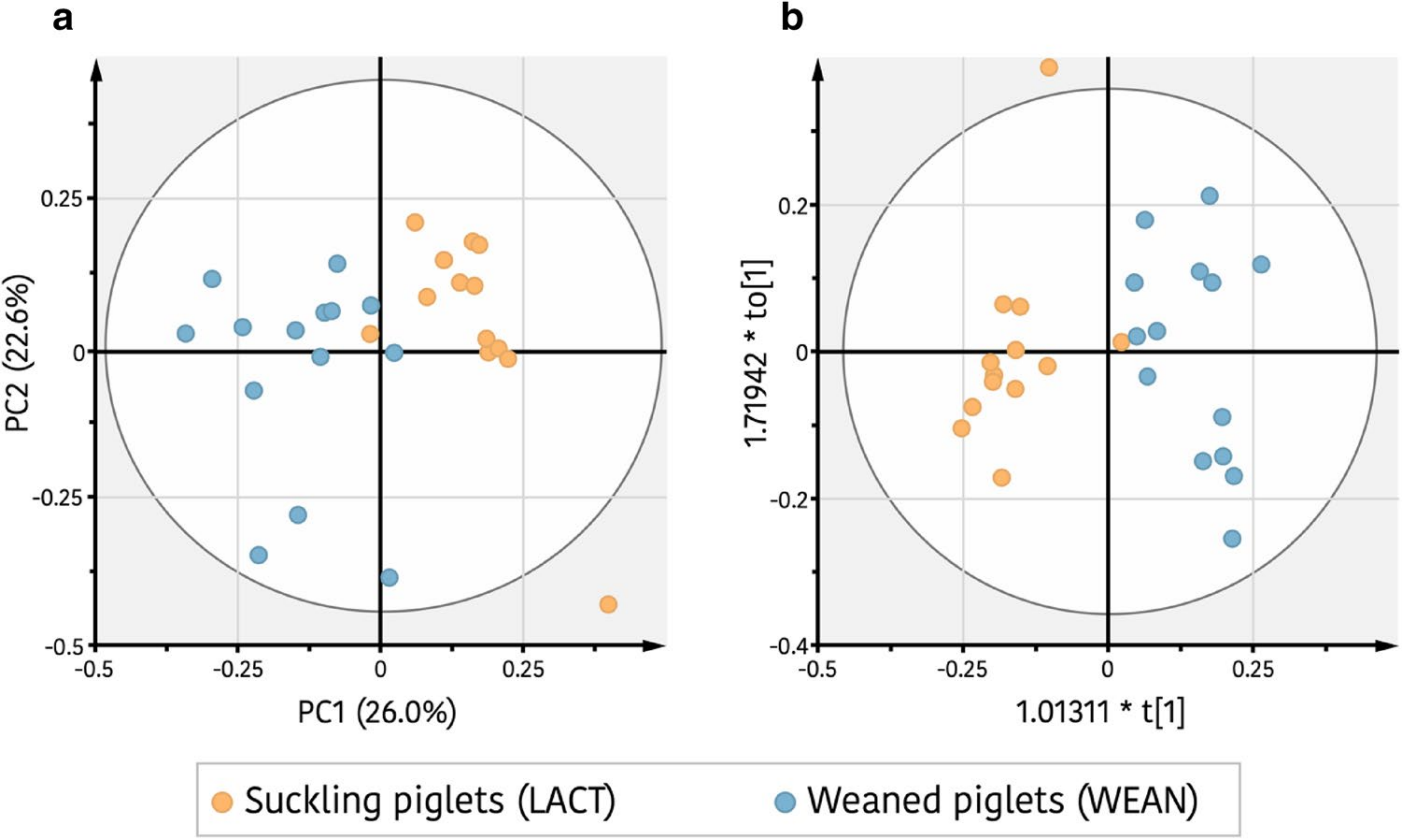
Weaning effect on serum metabolic profile of the piglets. (**a**) Principal components analysis (PCA) score plot of serum metabolites set from suckling piglets (orange) and weaned piglets (blue). (**b**) Orthogonal partial least squares discrimination analysis (OPLS-DA) score between suckling piglets (orange) and weaned piglets (blue).

To identify potential differences between serum metabolites profiles of pre- and post-weaning piglets, an untargeted metabolomics approach using ^1^H-NMR was also applied. In order to reduce the number of variables, a filtering of ^1^H-NMR bucket table was done by significant differences on Student’s t-test (*P*-value ≤ 0.2) between the integrated buck regions of suckling and weaned piglets. To evaluate the global metabolic profile of serum samples collected from piglets in both periods, a blinded to age groups study by principal component analysis (PCA) of ^1^H-NMR datasets was performed from the filtered ^1^H-NMR bucket table. Figure 5a shows a biplot representation of PCA [R^2^x_(cum)=_0.82, Q^2^_(cum)=_0.30] from the reduced data, in which a clear pattern of separation between suckling and weaned piglets along PC1 could be observed, indicating that both piglets’ groups were metabolically differentiated. To identify the key metabolites that influence in this grouping, a study taking account the age groups was made by an orthogonal partial least squares discriminant analysis (OPLS-DA) approach (Fig. 5b). The supervised OPLS-DA model [R^2^x_(cum)_ = 0.38, R^2^y_(cum)_ = 0.84, Q^2^_(cum)_ = 0.60] developed a perfect separation into the two clusters with high fitness R^2^ and accepted predictive ability Q^2^ parameters (R^2^y_(cum)_ and Q^2^_(cum)_ > 0.5). Moreover, the cross-model validation (Supplementary Fig. S2a) and the permutation test (100 times) (Supplementary Fig. S2c), both indicate that the developed OPLS-DA approach was positive and valid, confirming the distinction among both piglets’ groups. Also, a value of 1.0 for the area under the curve (AUC) corresponding to receiver operating characteristic (ROC) plot (Supplementary Fig. S3) indicated a strong discrimination power for the OPLS-DA classifier model. To find the most relevant ^1^H-NMR regions that contribute to the differentiation between suckling piglets from weaned piglets, an S-plot was performed (Supplementary Fig. S4). From this plot, the key ^1^H-NMR buckets that affect the discrimination were identified. These regions were also screened according to their corresponding variable importance in the projection (VIP) values of the OPLS-DA model. Fifteen from the total 250 spectral regions were found like the more contributing (Table 3), of which 12 regions had integral values that differed significantly between both groups (Student’s t-test *P*-value ≤ 0.05). The discriminant metabolites that showed higher levels in suckling piglets were choline, lipids (including triglycerides and fatty acids), LDL, alanine, isoleucine and probably also TMAO, whereas in weaned piglets those with higher levels were 3-hydroxybutyrate, ethanol, valine, and adipate (Table 3).

**Table 3:** Statistically significant key metabolites that differentiate serum of weaned piglets from suckling piglets.

^1^H Chemical shift ppm (Central bucket point)	Metabolite	Moieties	KEEG IDs	Weaned vs suckling		
				Fold Change^a^ weaning/lactation	*P*-value^b^	VIP^c^
3.22	Choline	${\text{N}} - ({\text{CH}}_{3} )_{3}$	C00114	0.7	0.0002	4.36
1.14	3-hydroxybutyrate	$\gamma {\text{CH}}_{3}$	–	6.6	0.0001	4.11
3.66	Ethanol Isoleucine	${\text{CH}}_{2}$ $\alpha {\text{CH}}$	C00469 C00407	1.4	0.0350	2.88
3.54	AA + Glucose			1.3	0.0385	2.82
1.22	Lipids^e^	$CH_{3} (CH_{2} )_{n}$	–	0.7	0.0127	2.04
0.82	LDL^f^	${\text{CH}}_{3}^{*} ({\text{CH}}_{2} )_{n} -$	–	0.6	0.0030	1.99
2.26	Valine	$\beta CH$	C00183	1.5	0.0044	1.63
1.50	Alanine	$\beta CH_{3}$	C00041	0.6	0.0209	1.57
0.98	Valine Isoleucine	$\gamma CH_{3}$	C00183 C00407	1.3	0.0300	1.56
1.02	Valine Isoleucine	$\gamma CH_{3}$	C00183 C00407	1.5	0.0018	1.42
2.22	Valine	$\beta CH$	C00183	1.7	0.0764	1.28
1.98	Isoleucine	$\beta CH$	C00407	0.7	0.0007	1.25

^a^Fold change was calculated by dividing the mean of normalized integral of each plasma metabolite in the former by the mean of normalized integral of each plasma in the latter. Fold change > 1 indicates that the metabolite was incremented, whereas fold change < 1 indicates the metabolite was reduced.

^b^*P*-values were derived from Student’s *t*-test.

^c^VIP value was derived from OPLS-DA with a threshold of 1.0

^d^TMAO: Trimethylamine-N-oxide.

^e^Lipids: Triglycerides and fatty acids.

^f^LDL: Low density lipoprotein.

### Integration of the omics technologies

Gene expression, caecal microbiota and metabolomics datasets were integrated by using the open-source software R^21^ v3.6.1. and the LinkHD^22^ package. The objective of this approach was to analyse these heterogeneous datasets to verify from a holistic point view that weaning was determinant defining different clusters of samples. Therefore, confirming our hypothesis, with the additional value to explore the connections (i.e.: correlations) between the gene expression, metabolomics and cecal microbial communities. Furthermore, the use of LinkHD allow us to highly the most informative variables within each dataset, as well as to identify which dataset was most relevant for the sample stratification. Although no model was applied in the statistical design, the samples were stratified into two clusters in a blind analysis, that aligned quite well with the groups of suckling and weaned piglets. The different ordination was mainly explained by the changes observed in piglet microbiota but also by the differential distribution of metabolites. Gene expression did not appear to contribute significantly for the cluster ordination probably due to the low number of input variables (52 genes) compared with the microbial and metabolites datasets. In total, 93 OTUs and 12 metabolites were found as discriminating between groups although the metabolites were not able to be identified. The discriminant relevant families included among others, *Fusobacteriaceae* (*P* = 0.0010), *Bacteroidaceae* (*P* = 0.0011), *Enterobacteriaceae* (*P* = 0.0012), *Lactobacillaceae* (*P* = 0.0013), *Erysipelotrichaceae* (*P* = 0.0138) and *Prevotellaceae* (*P* = 0.0424). Meanwhile, at genera level the significant discriminant genera were among others, *Fusobacterium* (*P* = 0.0010), *Bacteroides* (*P* = 0.0011), *Lactobacillus* (*P* = 0.0013), *Megasphaera* (*P* = 0.0014) and *Ruminococcus* (*P* = 0.0157).

**Figure 6:**
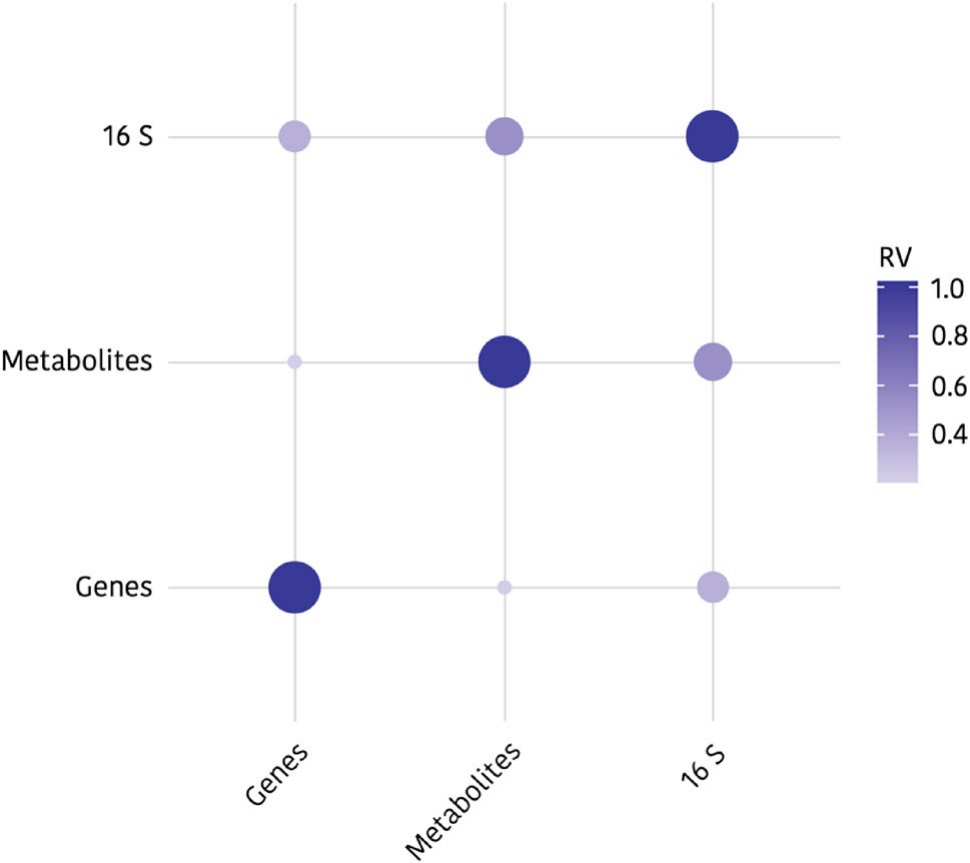
Correlation plot between gene expression (Genes), caecal microbiota (16 S) and metabolomic (Metabolites) datasets. The correlation between caecal microbiota and serum metabolome is much higher than with jejunal gene expression (Multivariate correlation values (RV): Genes & 16S = 0.37; Genes & Metabolites = 0.22; 16S & Metabolites = 0.53). Figure created by using open-source software R^21^ v3.5.3. (https://www.r-project.org/foundation/) and the LinkHD^22^ package (https://github.com/lauzingaretti/LinkHD).

In addition to the stratification in clusters and the identification of the most relevant variables, LinkHD also related the existing multivariate correlation (RV) between the different datasets (Fig. 6). Thus, it was observed that the strongest association was that between the metabolites and 16S (RV values: Genes & 16S = 0.37; Genes & Metabolites = 0.22; 16S & Metabolites = 0.53).

### Integration of gut microbiome and serum metabolome data

Considering the relevance of microbiota and metabolomic data in the LinkHD analysis, an additional integration approach was performed by correlating the caecal microbiota with the blood serum metabolome. The objective was to elucidate possible relationships between the intestinal microbiota and the animal metabolism, exploiting the wide range of response found around weaning. A multivariate analysis of the data was performed looking for significant correlations among the traits (Supplementary Table S2). The variables integrated were ^1^H-NMR bucket area regions of 0.04 ppm width (58) and bacteria family counts (22), obtained from nursing piglets (n = 14) and weaned piglets (n = 14). The 58 bucket regions, from the total of 250 regions of the spectra, were selected based on previous assigned soluble metabolites by Clausen et al.^23^ and He et al.^24,25^ The metabolites that showed significant Pearson correlation coefficients |r|≥ 0.37 (*P*-value ≤ 0.05) with the relative abundance of bacterial at family taxonomic level can be found in Supplementary Table S3.

**Figure 7:**
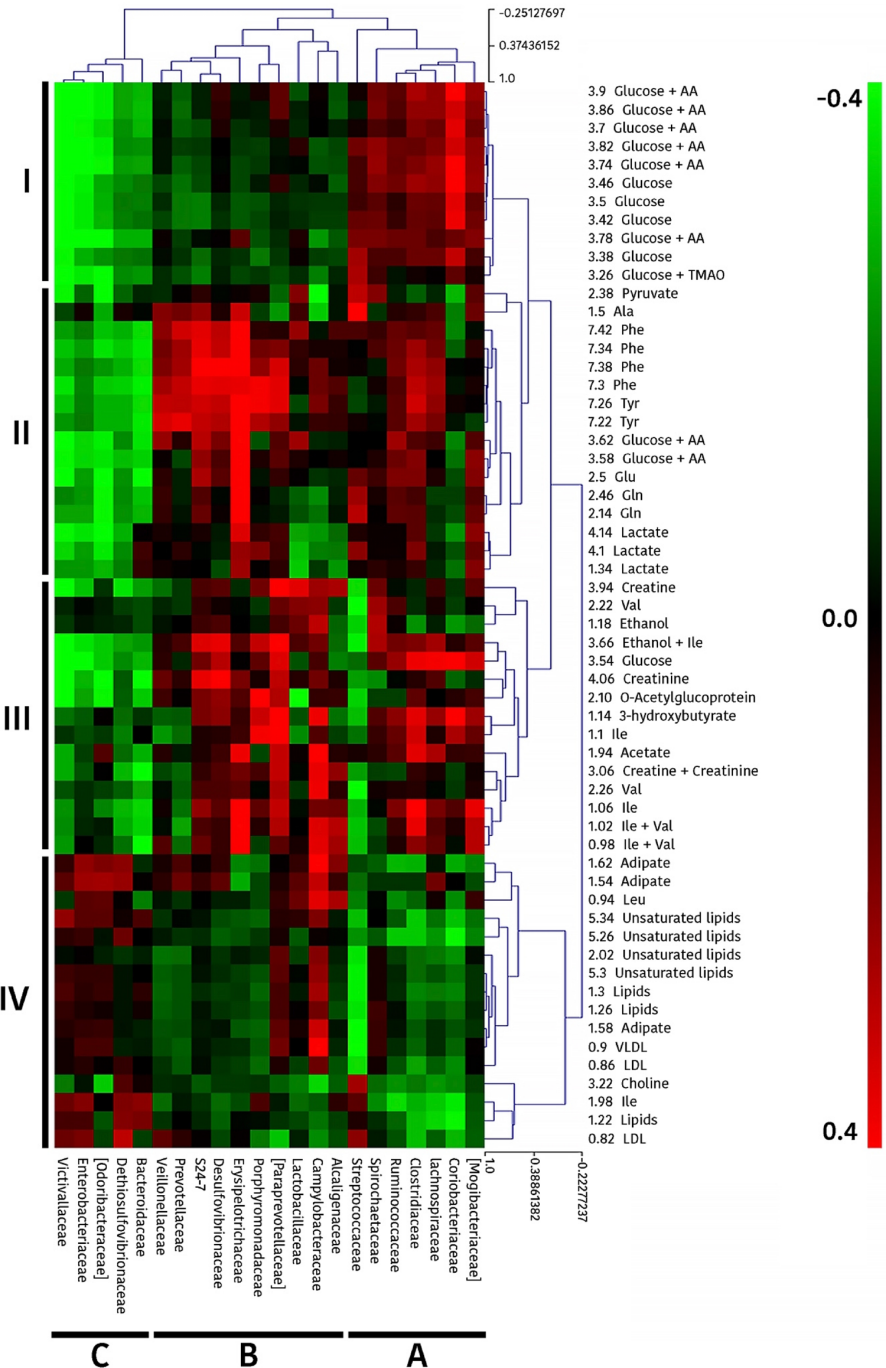
Heatmap showing the correlation analysis between gut microbiota and serum metabolome (^1^H-NMR bucket area regions) in piglets. Red or green spots indicate positive or negative Pearson correlations between variables, respectively, and the colour intensity is directly proportional to the correlation coefficient. Four clusters (designated as I, II, III or IV) are identified for metabolite buckets and 3 major clusters (named A, B and C) for bacterial families. Figure created by using open-source software R21 v3.5.3. (https://www.r-project.org/foundation/).

Furthermore, to better understand the putative relationships between gut microbiota and serum metabolites, a global analysis was performed by a hierarchical clustering of Pearson correlations coefficients (Fig. 7). This analysis evidenced four clusters for metabolite buckets (illustrated as cluster I-IV in Fig. 7) and 3 major clusters for bacteria families (illustrated as A, B and C). Interestingly, the clusters evidenced for metabolite buckets corresponded to certain chemical or metabolic categories. In this way, Cluster I included mainly glucose; Cluster II appears to be related to protein metabolism including aromatic (Tyr, Phe) and other amino acids (Ala, Gln, Glu) and glucose metabolism (glucose, pyruvate and lactate); Cluster III mostly included branched chain amino acids (Ile, Val) and also compounds related to energy metabolism (creatin/creatinine, 3-hydroxybutyrate and acetate) and Cluster IV included buckets mostly related to lipid metabolism (unsaturated lipids, lipids [triglycerides and fatty acids], LDL, VLDL, choline). Regarding microbial clusters, Cluster A conformed by *Mogibacteriaceae*, *Coriobacteriaceae*, *Lachnospiraceae*, *Clostridiaceae*, *Ruminococcaceae*, *Spirochaetaceae* and *Streptococcaceae* families, showed to be in general terms positively correlated with Cluster I and cluster III and negatively correlated with Cluster IV. Cluster B, including *Alcaligenaceae*, *Campylobacteraceae*, *Lactobacillaceae*, *Paraprevotellaceae*, *Porphyromonadaceae*, *Erysipelotrichaceae*, *Desulfovibrionaceae*, *S24-7*, *Prevotellaceae* and *Veillonellaceae*, differed from Cluster A in that Cluster B was negatively correlated with Cluster I and positively correlated with Cluster II. Finally, Cluster C, including *Bacteroidaceae*, *Dethiosulfovibrionaceae*, *Odoribacteraceae*, *Enterobacteriaceae* and *Victivallaceae* families, differed from Clusters A and B in that it was strongly negative correlated with cluster I, II and III, and weakly positive correlated with cluster IV.

## Discussion

The set of environmental, dietary and social changes to which piglets are subjected at weaning, together with their immaturity, both intestinal and immune, are source of relevant changes in the animal response and in the microbial ecosystem that symbiotically habit in their gastrointestinal tract^2,26,27^. It is nowadays recognized that these changes can be decisive for the appropriate development and growth of the animal^8^. A better understanding of these phenomena therefore appears as an essential tool to improve productivity and welfare of piglets. The aim of the present study was to evaluate the impact of weaning on caecal microbial colonization, the jejunal gene expression and the serum metabolome of the piglets to better understand the changes produced around weaning.

The high throughput sequencing of the 16S RNA gene showed that, in general terms, the diversity and community structure of caecal microbiota were in consonance with the predominant taxa described previously for healthy piglets^28^. The species richness and diversity of caecal microbiota were increased in piglets after weaning as reported by other studies^29–33^, where a continuous increase in alpha diversity of gut microbiota during weaning transition was observed. A higher diversity in the gut microbiota has been related to a more mature gut ecosystem and is in agreement with the concept of functional redundancy, which supports that additional taxa add redundancy to specific functions, helping the ecosystem to preserve its resilience and stability after environmental stresses^34,35^. Diversity results are, however, contradictory with other studies that have reported a decreased alpha diversity during the early period after weaning^36–38^, with a later increase from weaning to adulthood. This controversy could be due to differences between studies in the day samples were collected but also to differences in management and feeding practices or in the microbiological environment of the farm. In this sense, there may be also great differences between results obtained in controlled studies in experimental facilities and those carried out in conventional farms, where the stress to which the animals are subjected can be very different. Even between commercial farms, the husbandry conditions around weaning can be very according to the size of the farm, the environmental and sanitary conditions, and different country-associated regulations. In this regard results obtained in this study must be contextualized in a medium size farm, with a closed cycle system, weaning pigs at 25 days, and using medicated feed in the pre-starter period as metaphylaxis. Obviously, all these conditioning factors, and particularly the use of antimicrobials, can have a differential impact in other farms. When we use here the term *weaning*, its meaning goes further than the weaning itself, including also others conditioning factors associated. It is therefore important to be aware of the limitations of this study when translating their conclusions to other production systems. Regarding beta diversity, the interindividual Bray–Curtis distances between individuals decreased after weaning according to results reported by Chen et al.^31^, suggesting that gut microbiota structure of piglets trends to converge between animals after weaning.

*Bacteroidetes*, *Firmicutes* and *Proteobacteria* constituted the three predominant phyla in the caecal microbiota of piglets, both pre- and post-weaning, as reported in several studies^28,31,36,37,39^. The most predominant phylum was *Bacteroidetes* in suckling piglets and *Firmicutes* in weaned piglets. However, there is no consensus on the predominant phylum for piglets after weaning. Whereas some authors describe *Firmicutes*^31^ as the main phylum, others have reported *Bacteroidetes* as the most abundant in weaned piglets^32,36^. Again the different environments, diets and sampling date among experiments could explain this disparity. *Fusobacteria*, which was a predominant phylum during lactation, was significantly reduced in our study after weaning, as reported previously^29,31,32,36^. Some authors have suggested that the abundance of *Fusobacteria*, and therefore, *Fusobacterium*, is positively correlated with diarrheal swine diseases, such as the porcine epidemic diarrhoea and the new neonatal porcine diarrhoea^40,41^. Although a high individual variability is observed at genus level, *Bacteroides* and *Lactobacillus* showed remarkable decreases after weaning, in consonance with several previous studies^27,31,33,38,42^. Other genera, such as *Fusobacterium* and *Megasphaera* were also higher in suckling piglets, as stated by Chen et al.^31^ Nonetheless, some authors have reported increases in *Megasphaera* abundance after weaning^37,38,43^. The higher relative abundances of *Bacteroides* and *Lactobacillus* in suckling piglets have been correlated with a milk-oriented microbiome^42^. On the one hand, *Bacteroides* has been reported to use wide range of both milk oligosaccharides and host-derived glycans^44^. On the other hand, *Lactobacillus* is a well-known lactate producer by consuming simple milk sugars such as lactose^45^. Moreover, whereas *Fusobacterium* has been positively correlated to intestinal diseases^40,41,46^, *Lactobacillus* has been labelled as a major player in the establishment and the maintenance of the bacterial homeostasis after birth^47^. Therefore, the abrupt change to a solid cereal-based diet and the withdrawal of milk explain the decrease of the previously mentioned genera and the increase of butyrate-producing genera including *Roseburia*, *Ruminococcus*, and *Lachnospira*, among others^48^. Actually, and in consonance with other authors^31,33,43^, an increased abundance of *Roseburia* was observed in piglets after weaning. Moreover, other *Lachospiraceae* genera, such as *Lachnospira*, *Coprococcus* and *Dorea*, began to emerge after weaning. Although similar results were observed by Li et al.^37^, a decreased abundance of *Lachnospira* after weaning was reported by Frese et al.^42^. In agreement with other studies, *Ruminococcus* showed an increased abundance in weaned piglets^42^. The genera belonging to *Lachnospiraceae* and *Ruminococcaceae* families are adapted to metabolize a wide range of complex oligosaccharides and polysaccharides while producing short chain fatty acids (SCFA). Indeed, *Roseburia* is a major contributor in the metabolic network of carbohydrate utilization and production of butyrate^49^. Altogether, the higher abundance of *Roseburia*, *Ruminococcus*, *Coprococcus*, *Dorea* and *Lachnospira* genera in weaned piglets, adapted to digest resistant starches and dietary fibres to convert them to SCFA, show the quick microbial change of the piglets’ gut microbiota to cope with diets rich in complex carbohydrates, as these abundance shifts occur in a short period of time.

These taxonomic changes observed in the intestinal microbiota were consistent with the results obtained for the functional metabolic pathways of the caecal microbiota with PICRUST analysis. It was observed that the pathways related to different metabolic routes, mostly energy and protein metabolism, were more represented in the suckling piglets, whereas the pathways related to bacterial processes and environment and information processing, such as cell motility, membrane transport, sporulation or signal transduction were higher in weaned piglets. As it is well known, piglets experience anorexia and intestinal disorders shortly after weaning^2^. As a result, the gastrointestinal environment and functionality are severely affected. In this context, it seems consistent that metabolic pathways related to nutrient metabolism do not result in a competitive advantage within the gut microbiota ecosystem. Other studies have reported comparable results with a decrease in many metabolic pathways such as carbon fixation pathways, lipid biosynthesis proteins, citrate cycle (TCA cycle) and cofactor metabolism after weaning^37^, suggesting that the reduction in the nutrient availability related to post-weaning anorexia might be part of the microbiota adaptation process. On the other hand, the observed increase in the representativeness of pathways related to cell motility, membrane transport or signal transduction suggests that during the post-weaning phase those functions would confer competitive advantages to certain microbial groups. An adapted microbiota, as that found at the end of the lactating period, would prioritize the metabolic activity specialized in using a particular set of nutrients provided in this case by milk. However, shortly after weaning the microbiota would give priority to pathways related to adaptation to the new environment, among them, chemotaxis, motility, flagellar function, construction of the cytoskeleton and the activation of transporters. This transition can turn in a chance for opportunistic pathogens such as *E. coli* to proliferate and cause post-weaning diarrhoea. Actually, flagellar assembly and bacterial motility proteins, pathways exhibited between others by *E. coli*, showed higher representativeness after weaning, and could be regarded as an index of potential virulence factors^50^.

Regarding the possible impact of weaning on animal response and particularly on intestinal functionality, results from the OpenArray technology showed a significant decrease in the jejunal gene expression of *OCLN*, *CLDN4*, *MUC2* and *MUC13*. *OCLN* and *CLDN4* are transmembrane proteins of the tight junction (TJ)^51,52^. A downregulation in the expression of *OCLN* has also been reported associated to the weaning process^53^. Moreover, *MUC2* and *MUC13*, which encode a gel forming-mucin and a membrane-bound mucin respectively, were also downregulated in weaned piglets, reducing its protective effect on the intestinal epithelium. Actually the downregulation of MUC genes has also been associated with the presence of pathogenic bacteria, such as *ETEC*^54^.

Related to the immune function, a downregulation of IFNγR1 and an upregulation of PPARGC1α were observed after weaning. IFNγR1 is a cytokine receptor that encodes the ligand-binding chain of the gamma interferon receptor that has been reported to be upregulated by ETEC^55^. On the other hand, PPARGC1α, is an endogenous regulator of intestinal inflammation^56^, with an inhibitory effect on pro-inflammatory cytokines^57^. It has been reported to be upregulated during the transient suckling-weaning period in the jejunum of rats^58^. Therefore, the upregulation we observed in PPARGC1α in weaned piglets could be explained by weaning, constituting another factor to be considered in the downregulation of the inflammatory cytokines encoding genes mentioned above including IFNγR1.

Within the hormone/enzyme encoders category, *SI* and *HNMT*, two genes encoding digestive enzymes, were downregulated after weaning, whereas the *CCK* and *IGF1R*, two genes encoding digestive hormones, were upregulated. The enzyme encoded by the SI gene, is responsible for the digestion of dietary carbohydrates, whereas HNMT encodes a histamine-degrading enzyme. A downregulation in the expression of SI has been reported related to weaning^59^. As for the hormone encoders, *CCK* is involved in several activities such as satiety regulation, enzyme secretion, gut motility, and anxiety, whereas *IGF1R* is an important regulator of intestinal cell growth and differentiation^60^. Although in this study both genes showed increased expressions in weaned piglets, other authors have reported a downregulation after weaning^61,62^. A higher expression of CCK and *IGFR1* could be suggestive of a gut compensatory effect to increase the piglet digestion processes and stimulating the gut development after weaning. However, further research is required concerning the roles of *CCK* and *IGFR1* in these adaptive processes.

In the nutrient transporters category, a greater expression of *SLC16A1*—encoder of the monocarboxylate transporter 1 (*MCT1*) protein—was observed after weaning. The MCT1 mediates the absorption of lactate and microbial-derived SCFAs across cell membranes^63^ and has been reported to be upregulated by butyrate^64^. Therefore, an increased expression of SLC16A1 could be explained by an increased microbial fermentative activity after weaning with the production of lactate and other SCFAs. The *SLC15A1* and *SLC13A1* gene—encoders of the peptide transporter 1 (PEPT1) protein and sodium/sulphate symporters (NaS1), respectively—were downregulated in weaned piglets. *SLC15A1* has been related to absorption of protein digestion products^65^, whereas *SLC13A1* has been shown to play a role in the intestinal barrier function through sulfomucins^66^. Thus, lower expressions of these genes carry negative repercussions for gut health and nutrient digestion. In addition, some authors have reported decreases in the expression of *SLC15A1* and *SLC13A1* due the presence of pathogenic bacteria such as ETEC or *Lawsonia intracellularis*^67–69^. To end with nutrient transporters, two genes related to zinc transport also showed significant changes. While *SLC30A1*—encoder of the zinc transporter 1 (ZnT1) protein, related to the transport of zinc to the extracellular matrix—increased, the *SLC39A4* gene—encoder of the zinc transporter ZIO4, and related to zinc uptake from the gut lumen—decreased after weaning. *SLC39A4* has been shown to be downregulated by high dietary ZnO in piglets^70^ and, particularly, in piglets challenged with ETEC^71^, whereas *SLC30A1* has been shown to be upregulated by high dietary ZnO^70^. Therefore, these results are according to what would be expected by the introduction the weaning diet with pharmacological doses of ZnO. Finally, the *HSD11B1* gene, encoder of the 11β-Hydroxysteroid dehydrogenase type 1 enzyme, also known as cortisone reductase, and responsible for reducing cortisone to cortisol was upregulated in weaned piglets. A greater expression of *HSD11B1* after weaning could be related to a higher cortisol production, which is used as a marker of the levels of stress in piglets after weaning^72^.

Consistent with the changes observed in the jejunal gene expression around weaning, ^1^H-NMR results, also evidenced the relevant impact of weaning on the animal metabolomic response. Within 5 days between samplings, animals showed a quite different metabolomic pattern. The reduced serum levels of LDL, lipids (triglycerides and fatty acids), choline and the increased levels of the ketone body β-hydroxybutyrate after weaning, support the concept that weaning affects the metabolism of energy. Tucker et al.^73^ described β-hydroxybutyrate as a potential biomarker of metabolic stress as result of food and/or water restriction or deprivation. In fact, the stressful condition of weaning generally results in reduced feed intake during the first week after weaning since the piglets must adapt from a digestible and palatable maternal liquid milk to a solid dry diet. We can hypothesize that the significant increment of β-hydroxybutyrate observed 72 h after weaning could be the signal of an incipient (or previous) ketosis state considering fatty acids levels were not increased. Furthermore, it is known that serum levels of most amino acids undergo marked shifts in the neonatal period and in catabolic conditions. In this sense, reduced levels of the amino acid Ile after weaning could be due to its consumption as a precursor for ketonic bodies production that contribute to the increment of β-hydroxybutyrate. Likewise, lower levels of serum Ala in weaned piglets could be a consequence of its consumption during gluconeogenesis in the liver to provide glucose to extrahepatic cells and tissues^74^. However, it is worth to note that the metabolic profile of an organism is just a snapshot at a given time whereas metabolism is something dynamic and complex that can hardly be evaluated from a single picture.

The blinded integration of gene expression, metabolome and metagenome datasets with LinkHD R package confirmed the disparity between suckling and weaned piglets clustering them in two groups and also the higher correlation between caecal microbiota and serum metabolome compared to jejunal gene expression. The hierarchical clustering of Pearson correlation coefficients between the metabolomic changes and the shifts observed in microbiota, showed a clustering pattern (Fig. 7) that could suggest rational associations between gut microbiota structure and animal metabolic response. Four clusters of metabolite buckets (clusters I-IV) and 3 clusters of bacterial families (clusters A-C) could be identified. Interestingly, most of the metabolites that were increased (like 3-hydroxybutyrate, valine and ethanol) or decreased (among them choline, Ile, LDL and lipids) after weaning were within Cluster III or Cluster IV, respectively. Moreover, all bacteria families conforming Cluster A (except *Streptococcaceae*) increased after weaning while those conforming Cluster C decreased after weaning. Considering that Cluster A is positively correlated with Cluster III and negatively with Cluster IV, and Cluster C showed opposite sign correlations, results evidenced the relevant impact of weaning on the gut microbiota and piglet metabolic response.

The multivariate analysis performed to identify possible associations between metabolites and microbial groups showed several significant correlations (as shown in Supplementary Table S3). From those results we could hypothesize that the significant increment of *Coriobacteriaceae* (*P* = 0.0476) observed after weaning could be associated with the significant increment of serum 3-hydroxybutyrate and the decrease of lipids (r = 0.40 and r = − 0.39, respectively), while the significant reduction of *Streptococcaceae* (*P* = 0.0025) could be related with the significant elevated levels of valine and adipate (r = − 0.52 and r = − 0.42, respectively) and the significant lower levels of alanine (r = 0.48). In regard to the remaining correlations, other authors have also described significant positive correlations between the bacterial genera *Ruminococcus* and *Coprococcus*, from *Ruminococcaceae* and *Lachnospiraceae* families respectively, with glucose^75^. Moreover, it has been reported that *Firmicutes* genera such as *Streptococcus*, correlate positively with amino acids like valine, isoleucine and alanine^76^. Although the interplay between the gut microbiome and mammalian blood metabolites has been demonstrated^77^, it is difficult to establish if there is any causative effect in these correlations. There is still a long way to go to fully understand the interaction between the pig's intestinal microbiota and its impact on the serum metabolome.

## Conclusion

The present study evidenced the great changes that are produced at microbial, genetic and metabolic level in the piglet shortly after weaning. Caecal microbiota showed remarkable structural differences just 3 days after, with increases in alfa diversity and significant changes in taxonomic groups. The higher relative abundances of *Bacteroides* and *Lactobacillus* in suckling piglets correlated with a milk-oriented microbiome, whereas the higher abundances of *Roseburia*, *Ruminococcus*, *Coprococcus*, *Dorea* and *Lachnospira* genera in weaned piglets showed the quick adaptation of the piglets’ gut microbiota to cope with diets rich in complex carbohydrates. From the taxonomic changes observed in the intestinal microbiota we could also infer changes in functional metabolic pathways. Shortly after weaning the microbiota would give priority to pathways related to adaptation to the new environment rather than pathways related to different metabolic routes, as observed during lactation. At jejunal level a decrease was found in the gene expression of various barrier function genes (*OCLN*, *CLDN4*, *MUC2* and *MUC13*) and nutrient transport genes (such as *SLC15A1* and *SLC13A1*) revealing the negative impact of weaning on the intestinal functionality. Metabolomic approach evidenced the impact of weaning on the energy metabolism with increases in β-hydroxybutyrate levels and decreases in choline, LDL, triglycerides, fatty acids, alanine and isoleucine, suggesting an incipient ketosis state. Furthermore, the hierarchical clustering of Pearson correlations identified four metabolite clusters corresponding to specific chemical or metabolic categories suggesting potential causal relationships between microbiota and animal metabolism. In this sense, *Coriobacteriaceae*, significantly reduced after weaning, appears positively correlated to serum 3-hydroxybutyrate and negatively with lipids, while *Streptococcaceae*, significantly increased after weaning, appears negatively correlated to valine and adipate and positively to alanine.

In summary, the results of this study highlight the huge changes that occur in piglets raised in commercial farms shortly after weaning and show how microbiome and animal metabolism respond to it in a coordinated and an interdependent way evidencing the interplay between the gut microbiota and its host.

## Materials and methods

### Animals and experimental design

The housing, management, husbandry and slaughtering conditions of this experiment conformed to the European Union Guidelines (Directive 2010/63/EU). All experimental procedures used in this study were approved by the Animal and Human Experimental Ethical Committee of Universitat Autònoma de Barcelona (UAB) (permit code CEEAH 3817) and designed in compliance with the ARRIVE guidelines.

The trial was carried out in a commercial farm with a breeding stock of 1130 sows with 50–60 farrowing sows per batch in a closed cycle system. Sows in lactation and piglets until fattening were fed with commercial diets. Lactating and pre-starter diets were corn-soy based, but also incorporated other cereals like wheat and barley (multi-cereal diets). Sows’ and piglets’ diets included *Saccharomyces cerevisiae* NCYC Sc 47 (E1702) (10^9^ CFU/kg) and piglets’ pre-starter diets also included 6-phytase and endo-1–4-beta-xylanase. Ingredients and additives included in each formula and their chemical composition can be found in supplementary material (Supplementary Table S4). Pre-starter diets also included prescribed antimicrobials as metaphylaxis after first signs of disease in some individua to prevent the rest of the piglets from meningitis (Amoxicillin trihydrate, 250 mg/kg) and post-weaning diarrhoea (Oxytetracycline, (150 mg/kg) and ZnO (3100 mg/kg)). Farm vaccination guidelines included: *Mycoplasma*, circovirus and Aujeszky for piglets, and swine influenza, *Escherichia coli*, PRRS, porcine parvovirus (PPV) and erysipelas for breeding sows.

A total of forty-eight sows and their litters (average litter size 14.1 ± 0.1) were included in this study. These animals formed part of a previous published study^17^ in which sows were randomly allocated into two groups (24 sows per group) balanced by sow parity. One group was subjected to a conventional management in individual farrowing pens, whereas in the other group, piglet socialization was allowed by removing the separation fences between two sows from 14 days after delivery (for details see Saladrigas-García et al.^17^). The possible impact of these treatments on microbiota and animal response have been previously shown and discussed^17^.

Sows were fed twice a day with ad libitum water. Piglets were provided with ad libitum water and creep feed from two weeks of age. The piglets were weaned at 25 days of age and randomly mixed with other piglets into a total of 16 pens (40 piglets/pen, ca. 0.20 m^2^/animal). Piglets housed in each pen had received the same management during the suckling period. Weaners were offered ad libitum commercial feed and water.

### Sample extraction

Fourteen litters were sampled on two days before weaning (− 2 d, n = 14), and three days after weaning (+ 3 d, n = 14). Seven litters had received a conventional management and the other seven litters an enriched management. In each litter, one male piglet of the medium birth weight was selected per sampling day. The piglets were sedated with an intramuscular injection containing 20 mg/kg of ketamine (Ketamidor) and 2 mg/kg of xylazine (Xilagesic), and humanely euthanized with an overdose of pentobarbital. Blood samples were collected after opening the abdominal cavity directly from the caudal vena cava and serum was obtained by centrifugation for 15 min at 3500 rpm and stored at -80 °C. Jejunum tissue samples (1 cm^2^) were collected from mid-jejunum (1 m after duodenum), washed thoroughly with PBS, and preserved in 1 mL of RNAlater (Deltalab, Rubí, Spain). Caecal content was collected directly from the cecum and immediately frozen in dry ice. Tissue and caecal samples were kept at -20ºC until further analysis.

### 16S rRNA gene sequencing

Deoxyribonucleic acid (DNA) was extracted from 250 mg of each caecal sample using the QIAamp DNA Stool Mini Kit (Qiagen, Hilden, Germany) according to the manufacturer’s instructions following the optimization steps. DNA concentration and purity were checked with NanoDrop ND-1000 spectrophotometer (NanoDrop Technologies, Wilmington, DE, USA). For 16S rRNA gene high-throughput sequencing, amplicon libraries were prepared using Nextera XT Index Kits 16S V3–V4 Amplicon-Seq Kit (Illumina, San Diego, CA, USA). For sequencing on the MiSeq instrument, the generated libraries were placed in the reagent cartridge and loaded on the instrument along with the flow cell. The MiSeq Reagent Kit V2 (500-cycle) (Illumina, San Diego, CA, USA) was used. All subsequent steps were performed on the MiSeq Illumina instrument.

For sequencing data bioinformatics, the sequence reads generated were processed using Quantitative Insights Into Microbial Ecology (QIIME) version 1.9.1 software^78^. Open-reference OTU picking^79^ at 97% identity was performed with bacterial 16S GreenGenes (v. 13_8) reference database^80^. A detailed description of all further steps in the bioinformatic analysis is available in our previous publication^17^.

### Prediction of the functions of the microbial population

The predicted metagenome of caecal samples based on 16S rRNA gene sequencing data was analysed using Phylogenetic Investigation of Communities by Reconstruction of Unobserved States (PICRUSt v1.1.3)^18^ (http://picrust.github.io/picrust/) and the software STAMP v2.1.3^20^. (https://github.com/dparks1134/STAMP). For this, a closed reference OTU table was created in QIIME using filter_otus_from_otu_table.py script and the Greengenes reference database v13.5^80^. The generated OTU table was used as input for the 16S rRNA gene copy number normalization with the normalize_by_copy_number.py script. Functional predictions of Kyoto Encyclopedia of Genes and Genomes (KEGG) Orthologs (KOs) were generated using the predict_metagenomes.py script, which were then summarized into KEGG pathways at KO level 3 using the categorize_by_function.py script. The software STAMP was finally used to identify functional pathways differentially expressed.

### RNA extraction and gene expression study by qPCR

The expression of a total of 52 genes related to intestinal health was studied through a custom OpenArray plate (Applied Biosystems, Foster City, CA, USA). Full details of the method has been previously described by González-Solé et al^81^. The method had been optimized to quantify the expression of relevant genes in jejunum. Genes were selected based on previous knowledge and other reported works in the literature. GAPDH, ACTB, TBP and B2M were used as housekeeping genes. RNA was extracted from 100 mg of frozen jejunum tissue with the RiboPure kit (Ambion, Foster City, CA, USA) following the manufacturer’s protocol. A final cDNA volume of 6 μl from each sample was transferred per duplicate to a TaqMan OpenArray custom plate for gene expression and run in a QuantStudio 12 K Flex Real-Time PCR System (ThermoFisher Scientific, Waltham, MA, USA). Gene expression data analysis was performed as specified by González-Solé et al^81^. A list of the 52 genes included in the custom plate can be found in Supplementary information (Supplementary Table S1).

### Nuclear magnetic resonance (NMR) spectroscopy

Metabolic profiles by ^1^H-NMR spectroscopy were performed according to previous procedures^17^. Different endogenous metabolites were assigned from the ^1^H-NMR spectra by comparing chemical shifts and multiplicities of peaks to free databases like the Human Metabolome Data Base (HMDB)^82^, the Biological Magnetic Resonance Data Bank (BMRB)^83^ and published studies^23–25,84^. Briefly, 400 μL of blood serum were mixed with 200 μL of a saline buffer 0.9% NaCl (wt/vol) in D_2_O in the 5 mm NMR tube^85^, to obtain mixture containing about 30% D_2_O. The sample was subjected to spectral analysis at 14.1 T (600.13 MHz frequency for 1H) on a Bruker AVANCE II 600 spectrometer, equipped with a z-axis pulsed field gradient 5 mm triple channel probe (TBI), BACS 60 automatic sample changer and a BCU-Xtreme unit for temperature control. Proton spectra were acquired at 300.0 K. Further details about the acquisition of proton spectra and ^1^HNMR data pre-processing details have already been published^17^.

Each spectrum was pre-processed prior statistical analysis using TOPSPIN 3.6 (Bruker BioSpin, Germany). An exponential Fourier Transform (FT) using a line broadening factor of 0.3 was used. Each spectrum was aligned using lactate signal for calibration (1.33 ppm), automatic phase and baseline correction were applied with manual refinement when necessary. After that, spectral data set between 0.00 and 10.00 ppm was transferred to AMIX 3.9 package to automatically reduce it into integrated spectral regions of equal width (0.04 ppm), to exclude the spectral region between 4.78 and 4.66 ppm containing water signal and to normalize to total area. The final bucket table, containing 250 area regions of 0.04 ppm wide, was extracted and used for statistical analysis.

### Statistical analysis

The details of the biostatistical analysis of caecal microbiota, the statistical analysis of gene expression and nuclear magnetic resonance, and the integration of gene expression, metagenomics and metabolites can be found in Saladrigas-García et al. (2021)^17^.

In brief, metagenomics biostatistics was performed in open-source software R v3.5.3.^21^ (https://www.r-project.org/foundation/) using the phyloseq package^86^ as a support for QIIME in R. Cumulative sum scaling (CSS)^87^ normalization of raw counts and differential abundance analysis were performed following the metagenomeSeq package pipeline^88^. Relative abundances were used to plot taxon abundances and statistical significance was assumed at *P* < 0.05. For gene expression statistical analysis, open-source software R v3.5.3. was used. Two-way ANOVA was performed, and Benjamini–Hochberg false discovery rate (FDR) was used to adjust P-values. Statistical significance was assumed at FDR < 0.05. Regarding ^1^H-NMR statistical analysis, integral data from bucket table was imported to SIMCA 14.1 software for multivariate data analysis. The validity of the prediction model was assessed in function of the values of model fitness (R^2^) and predictive capacity (Q^2^) parameters. The more contributing ^1^H-NMR spectral bucket regions (0.04 ppm) for the discrimination between both groups were identified from a combination of VIP plot and S-plot analysis. Variables with VIP values ≥ 1 and located high up or low to the left corner of the S-plot were selected. The integration of gene expression, metagenomics and metabolites was performed by using the open-source software R v3.6.1. and following the LinkHD package^22^ pipeline (https://github.com/lauzingaretti/LinkHD).

Ultimately, for metabolome and microbiota correlation, a multivariate analysis of the data was performed to find statistically significant correlations. The Pearson correlation coefficients were calculated using InfoStat statistical software^89^. Hierarchical clustering of correlations coefficients between bacterial family counts and ^1^H-NMR bucket regions and a heatmap were prepared using Multiple Array Viewer (MeV) software (CCCB, Boston, USA)^90^, where red and green spots indicated, respectively, positive or negative Pearson correlations between variables.

In all of the previously mentioned analyses, significant differences were declared at *P* ≤ 0.05, while 0.05 < *P* ≤ 0.10 were considered near significant trends.

## Supplementary Information

Supplementary Information.

## Data Availability

The raw sequencing data employed in this article has been submitted to the NCBI’s sequence read archive (https://www.ncbi.nlm.nih.gov/sra); BioProject: PRJNA767391.
