# Supplementary material for: Understanding host-microbiota interactions in the commercial piglet around weaning
Source: Sci Rep. 2021 Dec 6;11:23488. doi: 10.1038/s41598-021-02754-6 (PMC8648723; doi:10.1038/s41598-021-02754-6)
Supplement: Supplementary file 1 — Supplementary Information. [file 41598_2021_2754_MOESM1_ESM.pdf]

Understanding host-microbiota interactions in the commercial piglet around weaning.

Saladrigas-García, M.[1], D'Angelo M.[1], Ko, H.L.[1], Nolis, P.[2], Ramayo-Caldas, Y.[3], Folch, J.M.[4,5], Llonch, P.[1], Solà-Oriol, D.[1], Pérez J.F.[1], Martín-Orúe, S.M.[1] *

[1] Animal Nutrition and Welfare Service (SNiBA), Department of Animal and Food Science, Universitat Autònoma de Barcelona (UAB), 08193 Bellaterra, Spain

[2] Nuclear Magnetic Resonance (SeRMN), Universitat Autònoma de Barcelona (UAB), 08193 Bellaterra, Spain

[3] Animal Breeding and Genetics Program, Institute for Research and Technology in Food and Agriculture (IRTA), Torre Marimon, 08140 Caldes de Montbui, Spain.

[4] Plant and Animal Genomics, Centre for Research in Agricultural Genomics (CRAG), CSIC-IRTA-UAB-UB Consortium, 08193 Bellaterra, Spain.

[5] Department of Animal and Food Science, Universitat Autònoma de Barcelona (UAB), 08193 Bellaterra, Spain.

* Corresponding author (susana.martin@uab.cat; Tel.: +34-93-581-1504)

Supplementary information

Supplementary tables

Supplementary Table S1. List of the 52 genes related to intestinal health included in the custom OpenArray plate and functional group to which they were assigned.

Gene abbreviation	Gene full name	Functional group
OCLN	Occludin	Intestinal barrier
ZO1	Zonula occludens 1	Intestinal barrier
CLDN1	Claudin-1	Intestinal barrier
CLDN4	Claudin-4	Intestinal barrier
CLDN15	Claudin-15	Intestinal barrier
MUC2	Mucin 2	Intestinal barrier
MUC13	Mucin 13	Intestinal barrier
TFF3	Trefoil factor 3	Intestinal barrier
TLR2	Toll-like receptor 2	Pattern recognition receptors (PRRs)
TLR4	Toll-like receptor 4	Pattern recognition receptors (PRRs)
IL1β	Interleukin 1 beta	Immune response
IL6	Interleukin 6	Immune response
IL8	Interleukin 8	Immune response
IL10	Interleukin 10	Immune response
IL17A	Interleukin 17	Immune response
IL22	Interleukin 22	Immune response
IFN-γ	Interferon gamma	Immune response
TNF-α	Tumor necrosis factor alpha	Immune response
TGF-β1	Transforming growth factor beta 1	Immune response
CCL20	Chemokine (C-C motif) ligand 20	Immune response
CXCL2	Chemokine (C-X-C motif) ligand 2	Immune response
IFNGR1	Interferon gamma receptor 1	Immune response
REG3G	Regenerating-islet derived protein 3 gamma	Immune response
PPARGC1α	Peroxisome proliferative activated receptor gamma, coactivator 1 alpha	Immune response
FAXDC2	Fatty acid hydrolase domain containing 2	Immune response
GBP1	Guanylate binding protein 1	Immune response
HSP27	Heat shock protein 27	Intestinal homeostasis
HSP70	Heat shock protein 70	Intestinal homeostasis

GPX2	Glutathione peroxidase 2	Digestive enzyme / hormone
SOD2	Superoxide dismutase	Digestive enzyme / hormone
ALPI	Intestinal alkaline phosphatase	Digestive enzyme / hormone
SI	Sucrase-isomaltase	Digestive enzyme / hormone
DAO1	Diamine oxidase	Digestive enzyme / hormone
HNMT	Histamine N-methyltransferase	Digestive enzyme / hormone
ANPEP	Aminopeptidase-N	Digestive enzyme / hormone
IDO1	Indoleamine 2,3-dioxygenase	Digestive enzyme / hormone
GCG	Glucagon	Digestive enzyme / hormone
CCK	Cholecystokinin	Digestive enzyme / hormone
IGF1R	Insulin-like growth factor 1 receptor	Digestive enzyme / hormone
PYY	Peptide YY	Digestive enzyme / hormone
SLC5A1	Solute carrier family 5 (sodium/glucose cotransporter) member 1	Nutrient transport
SLC16A1	Monocarboxylate transporter 1	Nutrient transport
SLC7A8	Solute carrier family 7 (amino acid transporter light chain, L System) member 8	Nutrient transport
SLC15A1	Solute carrier family 15 (oligopeptide transporter) member 1	Nutrient transport
SLC13A1	Solute carrier family 13 (sodium/sulfate symporters) member 1	Nutrient transport
SLC11A2	Solute carrier family 11 (proton-coupled divalent metal ion transporter) member 2	Nutrient transport
SLC30A1	Solute carrier family 30 (zinc transporter) member 1	Nutrient transport
SLC39A4	Solute carrier family 39 (zinc transporter) member 4)	Nutrient transport
CRHR1	Corticotropin releasing hormone receptor 1	Stress indicators
NR3C1-Grα	Glucocorticoid receptor	Stress indicators
HSD11B1	Hydroxysteroid (11-beta) dehydrogenase 1	Stress indicators
TBP	TATA-Box binding protein	Housekeeping

Supplementary Table S2. Pearson correlation coefficients between NMR bucket regions and bacterial families. Significant correlations are indicated in bold (p ≤ 0.05)

Variable	1	2	3	4	5	6	7	8	9	10	11	12	13	14	15	16	17	18	19	20	21	22	23	24	25	26	27	28	29	30	31	32	33	34	35	36	37	38	39	40	41	42	43	44	45	46	47	48	49	50	51	52	53	54	55	56	57	58	59	60	61	62	63	64	65	66	67	68	69	70	71	72	73	74	75	76	77	78	79	80
**1 (7.42 Phe)**	1
**2 (7.38 Phe)**	**0,86**	1,00
**3 (7.34 Phe)**	**0,96**	**0,91**	1,00
**4 (7.30 Phe)**	**0,75**	**0,86**	**0,85**	1,00
**5 (7.26 Tyr)**	**0,61**	**0,79**	**0,73**	**0,96**	1,00
**6 (7.22 Tyr)**	**0,77**	**0,87**	**0,81**	**0,91**	**0,93**	1,00
**7 (5.34 Unsaturated lipids)**	0,00	-0,13	-0,06	-0,22	-0,29	-0,26	1,00
**8 (5.30 Unsaturated lipids)**	0,08	0,02	0,07	-0,07	-0,17	-0,16	**0,91**	1,00
**9 (5.26 Unsaturated lipids)**	0,01	-0,10	0,03	-0,11	-0,12	-0,12	**0,68**	**0,60**	1,00
**10 (4.14 Lactate)**	0,20	0,01	0,24	0,12	0,08	0,08	-0,23	-0,22	0,06	1,00
**11 (4.10 Lactate)**	0,08	-0,05	0,13	0,12	0,10	0,05	-0,37	-0,34	-0,16	**0,90**	1,00
**12 (4.06 Creatinine)**	**0,59**	**0,63**	**0,63**	**0,70**	**0,63**	**0,59**	-0,09	0,06	0,11	0,22	0,12	1,00
**13 (3.94 Creatine)**	**0,41**	0,44	**0,42**	**0,42**	0,29	0,26	0,26	0,36	0,15	-0,03	-0,21	**0,53**	1,00
**14 (3.90 Glucose + AA)**	-0,18	-0,02	-0,11	0,11	0,18	0,09	**-0,72**	**-0,64**	**-0,50**	0,22	0,35	0,21	0,03	1,00
**15 (3.86 Glucose + AA)**	-0,11	0,02	-0,05	0,10	0,17	0,11	**-0,69**	**-0,61**	**-0,47**	0,26	0,33	0,24	0,11	**0,98**	1,00
**16 (3.82 Glucose + AA)**	-0,20	-0,18	-0,21	-0,10	-0,06	-0,08	**-0,64**	**-0,63**	**-0,50**	0,29	**0,39**	0,09	0,00	**0,93**	**0,93**	1,00
**17 (3.78 Glucose + AA)**	0,10	0,04	0,08	0,02	0,05	0,10	**-0,65**	**-0,67**	**-0,45**	**0,51**	**0,50**	0,19	0,00	**0,79**	**0,84**	**0,88**	1,00
**18 (3.74 Glucose + AA)**	-0,22	-0,15	-0,21	-0,05	0,01	-0,03	**-0,66**	**-0,62**	**-0,46**	0,23	0,33	0,08	-0,01	**0,96**	**0,96**	**0,97**	**0,83**	1,00
**19 (3.70 Glucose + AA)**	-0,23	-0,13	-0,20	0,00	0,07	0,01	**-0,72**	**-0,66**	**-0,57**	0,19	0,31	0,16	-0,04	**0,96**	**0,94**	**0,94**	**0,81**	**0,94**	1,00
**20 (3.66 Ethanol + Ile)**	0,30	**0,50**	**0,43**	**0,58**	**0,52**	**0,38**	-0,20	-0,03	-0,33	0,05	0,08	**0,52**	**0,57**	0,34	**0,38**	0,21	0,20	0,21	0,26	1,00
**21 (3.62 Glucose + AA)**	**0,62**	**0,48**	**0,58**	**0,37**	0,28	0,35	-0,11	-0,07	-0,07	**0,50**	0,30	**0,58**	0,36	0,03	0,15	0,13	**0,44**	0,04	0,04	**0,42**	1,00
**22 (3.58 Glucose + AA)**	**0,71**	**0,58**	**0,73**	**0,47**	**0,39**	**0,49**	-0,12	-0,09	0,02	**0,52**	0,31	**0,47**	0,35	-0,04	0,06	-0,03	**0,38**	-0,09	-0,07	0,23	**0,78**	1,00
**23 (3.54 Glucose)**	-0,18	0,13	-0,05	0,26	0,32	0,15	**-0,64**	**-0,49**	**-0,55**	0,01	0,15	0,28	0,14	**0,87**	**0,83**	**0,70**	**0,51**	**0,77**	**0,81**	**0,58**	-0,03	-0,15	1,00
**24 (3.50 Glucose)**	-0,29	-0,21	-0,27	-0,12	-0,03	-0,06	**-0,68**	**-0,66**	**-0,46**	0,20	0,31	0,02	-0,12	**0,95**	**0,94**	**0,95**	**0,81**	**0,98**	**0,95**	0,09	-0,05	-0,12	**0,72**	1,00
**25 (3.46 Glucose)**	-0,28	-0,13	-0,24	-0,08	-0,01	-0,07	**-0,67**	**-0,60**	**-0,59**	0,11	0,27	0,02	-0,03	**0,96**	**0,94**	**0,94**	**0,77**	**0,96**	**0,94**	0,27	-0,05	-0,16	**0,83**	**0,95**	1,00
**26 (3.42 Glucose)**	-0,28	-0,21	-0,27	-0,15	-0,07	-0,09	**-0,66**	**-0,65**	**-0,45**	0,22	0,33	-0,01	-0,10	**0,94**	**0,94**	**0,96**	**0,83**	**0,98**	**0,94**	0,08	-0,04	-0,10	**0,71**	**0,99**	**0,96**	1,00
**27 (3.38 Glucose)**	-0,22	-0,22	-0,27	-0,19	-0,14	-0,11	**-0,67**	**-0,67**	**-0,62**	0,24	**0,40**	-0,02	-0,19	**0,85**	**0,84**	**0,93**	**0,83**	**0,90**	**0,91**	0,06	0,05	-0,09	**0,62**	**0,92**	**0,91**	**0,92**	1,00
**28 (3.26 Glucose + TMAO)**	-0,11	-0,13	-0,14	-0,10	-0,05	-0,01	**-0,64**	**-0,66**	-0,30	**0,39**	**0,42**	0,15	-0,11	**0,84**	**0,84**	**0,90**	**0,87**	**0,89**	**0,86**	0,00	0,16	0,07	**0,53**	**0,90**	**0,81**	**0,91**	**0,87**	1,00
**29 (3.22 Choline)**	-0,17	-0,27	-0,18	-0,26	-0,21	-0,16	-0,22	-0,35	0,29	**0,52**	**0,43**	0,08	-0,30	0,32	0,33	**0,43**	**0,47**	**0,39**	0,32	-0,31	0,10	0,03	0,02	**0,41**	0,25	**0,43**	0,37	**0,66**	1,00
**30 (3.06 Creatine + Creatinine**	**0,62**	**0,67**	**0,67**	**0,63**	**0,51**	**0,53**	0,12	0,30	0,21	0,03	-0,11	**0,58**	**0,65**	-0,16	-0,12	-0,34	-0,18	-0,28	-0,24	0,28	0,30	**0,54**	-0,05	-0,31	-0,28	-0,31	-0,36	-0,26	-0,37	1,00
**31 (2.5 Glu)**	**0,59**	**0,49**	**0,61**	**0,42**	0,33	**0,39**	-0,25	-0,21	-0,10	**0,51**	**0,41**	**0,38**	0,16	0,09	0,15	0,10	**0,48**	0,07	0,05	0,29	**0,74**	**0,85**	-0,02	-0,01	0,00	0,02	0,06	0,18	0,07	0,37	1,00
**32 (2.46 Gln)**	**0,48**	**0,38**	**0,49**	0,30	0,28	0,37	-0,25	-0,28	-0,03	**0,50**	**0,39**	0,23	-0,04	0,01	0,05	0,04	**0,43**	0,03	-0,01	0,04	**0,63**	**0,79**	-0,14	-0,03	-0,07	0,01	0,04	0,20	0,17	0,27	**0,92**	1,00
**33 (2.38 Pyruvate)**	0,29	0,05	0,26	0,03	-0,09	-0,05	-0,08	-0,15	0,21	**0,61**	**0,47**	0,18	0,16	0,13	0,18	0,30	**0,49**	0,24	0,09	0,09	**0,51**	**0,45**	-0,10	0,12	0,07	0,19	0,12	**0,39**	**0,55**	-0,05	**0,60**	**0,51**	1,00
**34 (2.26 Val)**	0,19	0,23	0,24	0,25	0,18	0,11	**0,67**	**0,83**	**0,44**	-0,21	-0,34	0,25	**0,40**	**-0,46**	**-0,47**	**-0,58**	**-0,57**	**-0,54**	**-0,48**	0,20	0,04	0,08	-0,26	**-0,58**	**-0,52**	**-0,61**	**-0,67**	**-0,62**	**-0,49**	**0,43**	0,00	-0,13	-0,21	1,00
**35 (2.22 Val)**	0,21	0,18	0,21	0,08	-0,03	-0,03	**0,77**	**0,90**	**0,40**	-0,29	**-0,43**	0,15	**0,53**	**-0,51**	**-0,45**	**-0,50**	**-0,52**	**-0,51**	**-0,54**	0,29	0,12	0,02	-0,32	**-0,58**	**-0,48**	**-0,58**	**-0,61**	**-0,62**	**-0,48**	0,31	-0,09	-0,27	-0,08	**0,84**	1,00
**36 (2.14 Gln)**	**0,52**	0,37	**0,51**	0,30	0,25	0,35	-0,24	-0,27	-0,03	**0,59**	**0,50**	0,27	-0,03	0,03	0,07	0,08	**0,47**	0,05	0,00	0,01	**0,66**	**0,81**	-0,15	0,00	-0,05	0,04	0,09	0,23	0,20	0,27	**0,93**	**0,98**	**0,56**	-0,14	-0,28	1,00
**37 (2.1 O-Acetylglucoprotein)**	0,08	0,23	0,25	**0,56**	**0,62**	**0,40**	-0,20	-0,12	0,12	0,18	0,19	**0,47**	0,09	0,23	0,14	-0,02	0,00	0,05	0,17	0,30	-0,02	0,17	0,35	0,03	0,01	0,00	-0,16	0,00	-0,01	0,32	0,22	0,24	0,00	0,26	-0,15	0,20	1,00
**38 (2.02 Unsaturated lipids)**	0,13	0,03	0,14	-0,02	-0,11	-0,12	**0,89**	**0,95**	**0,73**	-0,05	-0,20	0,13	0,37	**-0,61**	**-0,60**	**-0,61**	**-0,59**	**-0,61**	**-0,65**	-0,07	-0,02	0,08	**-0,53**	**-0,65**	**-0,63**	**-0,63**	**-0,70**	**-0,59**	-0,21	0,37	-0,05	-0,10	0,02	**0,82**	**0,81**	-0,08	0,06	1,00
**39 (1.98 Ile)**	-0,09	-0,23	-0,08	-0,23	-0,21	-0,21	**0,45**	0,27	**0,82**	0,24	0,11	-0,09	-0,10	**-0,41**	**-0,43**	-0,36	-0,27	-0,37	**-0,47**	**-0,52**	-0,16	0,06	**-0,56**	-0,35	**-0,47**	-0,31	**-0,44**	-0,13	**0,50**	0,00	-0,01	0,14	0,37	0,07	-0,01	0,16	0,14	**0,48**	1,00
**40 (1.94 Acetate)**	0,27	**0,38**	0,34	0,38	**0,40**	**0,39**	-0,37	-0,33	-0,32	-0,03	0,03	0,12	0,12	0,35	**0,39**	0,22	0,27	0,25	0,31	0,26	0,16	0,29	0,28	0,28	0,31	0,27	0,26	0,19	-0,19	0,32	0,28	0,12	-0,14	-0,09	-0,18	0,15	0,01	-0,33	-0,36	1,00
**41 (1.62 Adipate)**	-0,18	-0,27	-0,21	-0,18	-0,25	-0,31	**0,58**	**0,41**	**0,49**	-0,31	-0,29	-0,04	0,02	-0,36	**-0,42**	-0,35	**-0,52**	-0,36	-0,33	-0,27	-0,31	-0,33	-0,32	-0,35	**-0,38**	-0,35	-0,35	-0,30	-0,06	0,05	-0,31	-0,35	-0,10	**0,39**	0,27	-0,31	0,07	**0,46**	**0,40**	-0,02	1,00
**42 (1.58 Adipate)**	0,02	-0,01	0,01	-0,08	-0,18	-0,19	**0,85**	**0,97**	**0,47**	-0,34	**-0,41**	0,04	0,35	**-0,55**	**-0,54**	**-0,56**	**-0,66**	**-0,55**	**-0,55**	0,06	-0,13	-0,20	-0,38	**-0,58**	**-0,50**	**-0,59**	**-0,59**	**-0,64**	**-0,44**	0,24	-0,28	**-0,40**	-0,24	**0,86**	**0,92**	**-0,39**	-0,14	**0,87**	0,09	-0,24	**0,47**	1,00
**43 (1.54 Adipate)**	-0,19	-0,20	-0,20	-0,08	-0,18	-0,32	**0,42**	**0,47**	0,15	**-0,46**	-0,29	0,00	0,03	-0,28	-0,35	-0,32	**-0,61**	-0,32	-0,27	0,01	-0,37	**-0,57**	-0,05	-0,32	-0,23	-0,34	-0,27	**-0,45**	**-0,41**	0,02	**-0,49**	**-0,62**	-0,36	**0,39**	**0,39**	**-0,56**	-0,01	0,35	-0,05	-0,12	**0,71**	**0,58**	1,00
**44 (1.5 Ala)**	0,29	0,00	0,23	0,00	-0,09	-0,04	-0,16	-0,30	0,15	**0,62**	**0,58**	0,04	-0,24	-0,07	-0,06	0,08	0,31	0,01	-0,09	-0,28	**0,39**	**0,44**	-0,27	-0,02	-0,12	0,02	0,08	0,21	**0,43**	-0,03	**0,56**	**0,59**	**0,70**	-0,36	**-0,39**	**0,68**	0,02	-0,11	**0,44**	-0,03	0,06	**-0,42**	-0,18	1,00
**45 (1.34 Lactate)**	0,02	-0,20	0,05	-0,06	-0,15	-0,21	0,12	0,12	0,19	**0,80**	**0,82**	0,02	-0,12	-0,03	-0,06	0,04	0,14	-0,02	-0,07	-0,07	0,26	0,24	-0,15	-0,05	-0,09	-0,03	0,04	0,07	0,25	-0,01	0,31	0,30	**0,43**	0,03	-0,06	**0,42**	0,12	0,25	0,31	-0,15	0,10	0,02	0,04	**0,61**	1,00
**46 (1.3 Lipids)**	0,00	-0,08	-0,03	-0,17	-0,27	-0,25	**0,93**	**0,98**	**0,57**	-0,18	-0,30	-0,04	0,29	**-0,64**	**-0,62**	**-0,60**	**-0,64**	**-0,62**	**-0,64**	-0,08	-0,09	-0,12	**-0,52**	**-0,64**	**-0,59**	**-0,63**	**-0,63**	**-0,64**	-0,31	0,18	-0,23	-0,30	-0,14	**0,81**	**0,87**	-0,28	-0,18	**0,93**	0,27	-0,33	**0,47**	**0,96**	**0,46**	-0,28	0,17	1,00
**47 (1.26 Lipids)**	0,07	-0,01	0,05	-0,12	-0,21	-0,20	**0,91**	**0,99**	**0,62**	-0,19	-0,32	0,03	0,36	**-0,62**	**-0,59**	**-0,59**	**-0,62**	**-0,60**	**-0,66**	-0,02	-0,04	-0,08	**-0,50**	**-0,64**	**-0,58**	**-0,63**	**-0,66**	**-0,63**	-0,30	0,23	-0,20	-0,28	-0,08	**0,80**	**0,91**	-0,26	-0,17	**0,94**	0,29	-0,36	**0,39**	**0,95**	**0,43**	-0,27	0,13	**0,98**	1,00
**48 (1.22 Lipids)**	-0,01	-0,17	-0,04	-0,25	-0,24	-0,17	**0,58**	**0,44**	**0,80**	0,23	0,02	-0,06	-0,04	**-0,44**	**-0,42**	-0,32	-0,24	-0,37	**-0,47**	-0,36	0,04	0,06	**-0,60**	**-0,38**	**-0,48**	-0,34	**-0,46**	-0,14	**0,52**	-0,17	-0,06	0,02	**0,39**	0,25	0,30	0,03	-0,10	**0,56**	**0,80**	**-0,41**	0,27	0,31	-0,13	0,22	0,23	**0,48**	**0,51**	1,00
**49 (1.18 Ethanol)**	0,32	0,36	0,32	0,22	0,16	0,22	**0,55**	**0,59**	0,31	-0,32	**-0,47**	0,18	**0,50**	-0,37	-0,29	**-0,38**	-0,33	**-0,38**	**-0,41**	**0,39**	0,19	0,13	-0,26	**-0,46**	**-0,38**	**-0,46**	**-0,54**	**-0,44**	-0,36	0,30	0,05	-0,10	0,01	**0,63**	**0,80**	-0,15	-0,12	**0,54**	-0,05	0,11	0,19	**0,62**	0,09	**-0,39**	-0,27	**0,59**	**0,62**	0,31	1,00
**50 (1.14 3-hydroxybutyrate)**	0,04	**0,45**	0,21	**0,39**	**0,39**	0,25	-0,07	0,16	-0,24	-0,35	-0,26	0,16	**0,37**	0,21	0,18	-0,05	-0,16	0,04	0,07	**0,68**	-0,08	-0,07	**0,54**	-0,01	0,22	-0,01	-0,12	-0,24	**-0,51**	0,30	-0,02	-0,19	-0,33	0,34	0,34	-0,24	0,20	0,06	**-0,43**	0,29	-0,23	0,25	0,14	**-0,56**	-0,33	0,09	0,15	-0,37	0,34	1,00
**51 (1.1 Ile)**	0,13	**0,48**	0,28	0,36	**0,40**	0,36	-0,02	0,21	-0,06	-0,17	-0,16	0,08	0,28	0,12	0,12	-0,10	-0,10	0,01	-0,02	**0,44**	-0,09	0,05	0,30	-0,03	0,12	-0,02	-0,18	-0,16	-0,25	0,24	0,03	-0,07	-0,20	0,34	0,32	-0,12	0,13	0,14	-0,19	0,14	**-0,41**	0,22	-0,20	**-0,51**	-0,32	0,14	0,21	-0,01	0,34	**0,82**	1,00
**52 (1.06 Ile)**	**0,42**	**0,56**	**0,52**	**0,52**	**0,46**	**0,41**	0,08	0,30	0,06	0,00	-0,16	0,27	**0,39**	-0,19	-0,17	-0,37	-0,20	-0,29	-0,24	0,32	0,23	**0,43**	0,02	-0,34	-0,27	-0,36	**-0,45**	**-0,39**	**-0,43**	**0,60**	**0,41**	0,26	-0,01	**0,65**	**0,42**	0,23	0,37	0,33	-0,13	0,17	-0,13	0,31	-0,10	-0,12	-0,09	0,24	0,27	-0,08	0,34	**0,50**	**0,57**	1,00
**53 (1.02 Ile + Val)**	**0,41**	**0,50**	**0,50**	**0,48**	**0,42**	0,36	0,05	0,26	0,05	-0,01	-0,17	0,26	0,36	-0,19	-0,16	-0,37	-0,19	-0,31	-0,21	0,23	0,24	**0,47**	-0,04	-0,31	-0,28	-0,33	**-0,41**	**-0,38**	**-0,47**	**0,67**	**0,37**	0,23	-0,10	**0,61**	0,35	0,21	0,34	0,29	-0,14	0,35	-0,04	0,28	-0,04	-0,09	-0,08	0,20	0,21	-0,16	0,27	**0,41**	**0,41**	**0,93**	1,00
**54 (0.98 Ile + Val)**	0,35	0,30	**0,41**	0,37	0,26	0,19	0,17	0,27	0,17	0,03	-0,14	0,28	0,30	-0,31	-0,29	**-0,42**	-0,26	**-0,39**	-0,28	0,09	0,26	**0,44**	-0,17	**-0,40**	**-0,42**	**-0,43**	**-0,44**	**-0,40**	**-0,42**	**0,65**	**0,39**	0,26	-0,01	**0,58**	0,30	0,27	**0,39**	0,36	-0,01	0,25	0,30	0,29	0,18	0,15	0,13	0,24	0,22	-0,15	0,18	0,09	-0,06	**0,74**	**0,84**	1,00
**55 (0.94 Leu)**	0,09	-0,08	0,09	0,03	-0,13	-0,20	**0,42**	0,37	**0,40**	-0,05	-0,14	0,13	0,35	-0,37	-0,36	-0,37	**-0,39**	**-0,38**	-0,36	-0,09	0,01	0,11	-0,30	**-0,40**	**-0,43**	**-0,40**	**-0,38**	-0,35	-0,23	**0,47**	0,05	-0,05	0,08	0,37	0,28	-0,01	0,15	**0,46**	0,29	0,08	**0,72**	0,37	**0,53**	0,24	0,26	0,36	0,33	0,02	0,11	-0,18	**-0,45**	0,17	0,30	**0,69**	1,00
**56 (0.9 VLDL)**	0,00	-0,05	0,01	-0,06	-0,18	-0,24	**0,88**	**0,93**	**0,55**	-0,24	-0,35	0,02	**0,39**	**-0,59**	**-0,58**	**-0,60**	**-0,68**	**-0,60**	**-0,61**	0,04	-0,10	-0,13	**-0,41**	**-0,64**	**-0,57**	**-0,64**	**-0,65**	**-0,68**	**-0,41**	0,31	-0,21	-0,33	-0,15	**0,83**	**0,86**	-0,31	-0,05	**0,90**	0,21	-0,19	**0,58**	**0,94**	**0,59**	-0,27	0,15	**0,94**	**0,92**	0,33	**0,59**	0,18	0,08	0,31	0,30	**0,43**	**0,59**	1,00
**57 (0.86 LDL)**	-0,03	-0,17	-0,03	-0,17	-0,28	-0,33	**0,84**	**0,85**	**0,78**	-0,02	-0,18	0,07	0,25	**-0,60**	**-0,59**	**-0,58**	**-0,61**	**-0,57**	**-0,64**	-0,15	-0,05	-0,08	**-0,50**	**-0,61**	**-0,62**	**-0,60**	**-0,65**	**-0,54**	-0,09	0,24	-0,16	-0,20	0,07	**0,66**	**0,69**	-0,17	0,00	**0,90**	**0,52**	**-0,39**	**0,60**	**0,79**	**0,52**	0,03	0,35	**0,85**	**0,86**	**0,55**	**0,41**	-0,08	-0,13	0,13	0,11	0,34	**0,63**	**0,88**	1,00
**58 (0.82 LDL)**	0,01	-0,22	-0,06	-0,25	-0,29	-0,20	0,32	0,10	**0,63**	0,14	0,01	-0,01	-0,24	**-0,40**	**-0,40**	-0,27	-0,17	-0,30	**-0,40**	**-0,48**	0,10	0,10	**-0,53**	-0,32	**-0,46**	-0,30	-0,27	-0,01	**0,48**	-0,09	0,11	0,28	0,36	-0,09	-0,07	0,27	-0,05	0,24	**0,69**	-0,31	**0,45**	-0,02	0,05	**0,54**	0,27	0,12	0,13	**0,59**	-0,04	**-0,62**	**-0,57**	-0,33	-0,35	0,03	**0,38**	0,12	**0,47**	1,00
**59 (Mogibacteriaceae)**	-0,02	0,01	0,07	0,04	0,01	-0,10	-0,18	-0,08	-0,21	0,22	0,16	-0,07	0,14	0,24	0,21	0,22	0,21	0,23	0,17	0,14	0,13	0,22	0,33	0,15	0,24	0,18	0,11	0,04	-0,10	0,03	0,21	0,13	0,14	0,05	0,01	0,16	0,11	-0,02	-0,13	0,09	-0,21	-0,08	-0,12	0,17	0,21	-0,08	-0,06	-0,14	-0,17	0,25	0,16	0,37	0,29	0,29	0,09	0,01	0,00	-0,15	1,00
**60 (Odoribacteraceae)**	-0,31	-0,24	-0,36	-0,32	-0,28	-0,28	0,14	0,08	-0,03	**-0,44**	-0,37	**-0,41**	-0,08	**-0,38**	**-0,38**	**-0,38**	**-0,50**	**-0,39**	-0,34	-0,32	**-0,38**	-0,36	-0,32	-0,28	-0,27	-0,29	-0,23	**-0,40**	-0,32	0,00	**-0,46**	**-0,39**	**-0,53**	-0,10	-0,04	**-0,39**	**-0,39**	-0,05	0,00	0,13	0,20	0,09	0,26	-0,27	-0,29	0,09	0,03	-0,13	-0,05	0,01	-0,09	-0,21	-0,04	-0,06	0,15	0,11	0,01	-0,10	-0,30	1,00
**61 (Paraprevotellaceae)**	0,06	0,34	0,20	0,36	0,36	0,23	0,06	0,14	0,04	0,11	0,10	0,30	**0,44**	0,15	0,15	-0,03	-0,07	-0,02	0,05	**0,54**	0,13	0,14	0,35	-0,06	0,08	-0,05	-0,09	-0,12	-0,15	0,31	0,08	-0,06	-0,16	0,27	0,16	-0,04	0,28	0,15	-0,04	0,31	0,01	0,15	0,05	-0,24	0,07	0,11	0,11	-0,08	0,11	**0,60**	**0,49**	0,31	0,28	0,14	0,08	0,18	0,05	-0,31	0,14	0,10	1,00
**62 (Alcaligenaceae)**	0,12	0,03	0,04	0,13	0,09	0,08	-0,04	-0,07	-0,01	-0,17	-0,19	0,05	0,27	-0,13	-0,14	-0,11	-0,14	-0,12	-0,05	-0,17	-0,05	0,03	-0,20	-0,09	-0,15	-0,10	-0,11	-0,06	-0,15	0,23	-0,06	-0,05	-0,03	0,07	-0,09	-0,03	0,09	-0,05	0,07	0,06	0,21	-0,03	0,09	-0,03	-0,24	-0,09	-0,12	-0,10	-0,16	-0,18	-0,10	0,17	0,29	0,26	0,27	-0,06	-0,13	-0,12	-0,13	**0,40**	0,03	1,00
**63 (Bacteroidaceae)**	-0,35	-0,35	-0,37	**-0,38**	-0,35	-0,37	0,12	0,00	0,00	0,01	0,10	**-0,53**	-0,25	-0,29	-0,30	-0,23	-0,28	-0,26	-0,29	-0,24	-0,36	-0,33	-0,29	-0,20	-0,19	-0,18	-0,18	-0,24	0,03	**-0,40**	**-0,40**	-0,31	-0,06	-0,26	-0,12	-0,29	-0,32	-0,05	0,22	-0,28	-0,07	-0,05	0,01	-0,01	0,08	0,07	0,03	0,19	-0,12	-0,08	0,00	-0,34	-0,33	**-0,43**	-0,20	-0,04	0,00	-0,10	-0,24	**0,56**	-0,07	0,07	1,00
**64 (Campylobacteraceae)**	-0,03	0,17	0,04	0,17	0,19	0,09	0,20	0,23	0,02	-0,24	-0,20	0,18	0,25	0,02	0,01	-0,14	-0,24	-0,13	0,00	0,18	-0,06	0,00	0,11	-0,09	-0,02	-0,10	-0,10	-0,20	-0,34	**0,37**	-0,08	-0,22	**-0,43**	**0,38**	0,22	-0,18	0,08	0,21	-0,14	**0,65**	**0,42**	0,32	0,31	-0,32	-0,09	0,26	0,18	-0,18	0,30	0,38	0,13	0,18	0,35	0,36	0,36	**0,40**	0,17	-0,22	-0,02	0,37	**0,51**	0,05	-0,11	1,00
**65 (Clostridiaceae)**	0,14	0,27	0,25	0,30	0,33	0,26	-0,32	-0,17	-0,32	0,14	0,20	0,06	-0,06	0,27	0,23	0,16	0,17	0,20	0,20	0,20	0,13	0,21	**0,38**	0,17	0,25	0,16	0,17	0,03	-0,19	0,08	0,20	0,11	-0,15	0,06	-0,07	0,14	0,15	-0,17	-0,28	0,36	-0,28	-0,14	-0,08	0,08	0,09	-0,21	-0,18	-0,30	-0,23	0,36	0,33	**0,40**	0,37	0,22	-0,16	-0,16	-0,26	-0,30	**0,69**	-0,29	0,29	-0,14	-0,36	0,11	1,00
**66 (Coriobacteriaceae)**	-0,21	-0,01	-0,15	-0,03	0,03	-0,03	-0,31	-0,18	**-0,39**	-0,23	-0,13	-0,16	-0,08	**0,42**	0,33	0,33	0,17	**0,40**	0,36	0,06	-0,21	-0,18	**0,55**	0,36	**0,49**	**0,38**	0,31	0,15	-0,21	-0,10	-0,12	-0,10	-0,29	-0,09	-0,10	-0,12	0,06	-0,23	-0,37	0,08	-0,27	-0,13	0,00	-0,20	-0,18	-0,21	-0,17	**-0,39**	-0,24	**0,40**	0,27	0,13	0,05	-0,02	-0,22	-0,16	-0,22	-0,29	**0,72**	-0,19	0,05	-0,24	-0,33	-0,01	**0,63**	1,00
**67 (Desulfovibrionaceae)**	0,30	**0,41**	0,31	0,36	0,31	0,28	-0,12	-0,07	-0,15	-0,04	-0,05	**0,48**	0,14	0,09	0,07	0,00	0,01	-0,01	0,05	**0,37**	0,19	0,12	0,29	-0,06	0,01	-0,08	-0,06	-0,05	-0,09	0,13	0,14	-0,01	-0,01	0,12	0,03	0,02	0,23	-0,08	-0,17	0,10	0,08	-0,03	0,15	0,05	-0,12	-0,10	-0,08	-0,15	0,12	0,22	0,06	0,14	0,09	0,09	-0,05	-0,06	-0,08	-0,11	0,12	-0,10	0,19	-0,05	-0,13	0,15	0,22	0,02	1,00
**68 (Dethiosulfovibrionaceae)**	-0,28	-0,28	-0,32	-0,33	-0,28	-0,28	0,03	-0,03	0,17	-0,28	-0,22	-0,14	-0,36	-0,26	-0,26	-0,22	-0,28	-0,25	-0,23	-0,26	-0,22	-0,30	-0,22	-0,19	-0,22	-0,20	-0,19	-0,22	0,13	-0,29	-0,23	-0,22	-0,16	-0,15	-0,10	-0,23	-0,15	-0,08	0,20	-0,17	0,22	-0,03	0,24	0,04	-0,20	-0,03	-0,03	0,21	-0,06	-0,17	-0,20	-0,29	-0,27	-0,18	0,01	-0,05	0,08	0,30	-0,24	**0,41**	-0,15	-0,01	0,16	-0,08	-0,27	-0,15	0,15	1,00
**69 (Enterobacteriaceae)**	-0,19	-0,19	-0,25	-0,21	-0,20	-0,19	0,14	0,08	0,09	-0,36	-0,25	-0,29	-0,31	**-0,45**	**-0,48**	**-0,40**	**-0,48**	**-0,41**	**-0,39**	-0,36	-0,34	-0,34	**-0,40**	-0,37	**-0,38**	-0,37	-0,28	-0,37	-0,03	-0,13	-0,25	-0,24	-0,16	-0,07	-0,01	-0,26	-0,26	0,01	0,21	-0,10	0,23	0,08	0,25	0,03	-0,24	0,07	0,05	0,11	-0,03	-0,14	-0,12	-0,07	-0,06	0,00	0,14	0,11	0,06	0,19	-0,28	**0,43**	-0,27	0,12	0,28	0,01	-0,18	-0,23	-0,19	**0,54**	1,00
**70 (Erysipelotrichaceae)**	**0,40**	**0,42**	**0,47**	0,37	**0,40**	**0,40**	-0,14	-0,10	-0,13	0,35	0,24	0,19	-0,07	-0,04	-0,03	-0,09	0,14	-0,11	-0,08	0,13	**0,52**	**0,57**	0,01	-0,14	-0,11	-0,14	-0,08	-0,11	-0,09	0,18	**0,49**	**0,46**	0,02	0,14	-0,01	**0,49**	0,13	-0,04	-0,12	**0,38**	-0,21	-0,14	-0,27	0,32	0,25	-0,09	-0,10	-0,01	0,00	0,09	0,10	**0,42**	**0,43**	**0,41**	-0,05	-0,07	-0,10	0,01	**0,56**	-0,27	0,22	-0,21	-0,36	0,19	**0,73**	0,31	0,24	-0,17	-0,21	1,00
**71 (Lachnospiraceae)**	0,16	0,23	0,18	0,25	0,23	0,19	-0,18	-0,13	-0,24	-0,09	-0,04	0,13	0,10	0,23	0,23	0,20	0,12	0,24	0,17	0,26	0,10	0,00	**0,39**	0,16	0,23	0,15	0,17	0,01	-0,24	0,04	0,05	-0,10	-0,06	-0,01	0,04	-0,07	0,00	-0,19	-0,32	0,27	-0,03	-0,07	0,18	0,12	-0,06	-0,19	-0,13	-0,34	-0,05	0,24	0,05	0,19	0,13	0,15	0,03	-0,07	-0,15	-0,15	**0,48**	-0,28	0,11	-0,22	**-0,42**	0,02	**0,74**	**0,50**	**0,38**	-0,10	-0,04	**0,45**	1,00
**72 (Lactobacillaceae)**	0,24	0,07	0,08	-0,01	-0,11	-0,03	0,07	-0,01	-0,14	-0,26	-0,30	0,06	0,36	-0,13	-0,05	0,07	0,05	-0,01	-0,06	0,12	0,25	0,05	-0,13	-0,08	-0,06	-0,07	0,02	-0,07	-0,20	-0,01	0,01	-0,15	0,21	-0,08	0,21	-0,13	**-0,40**	-0,10	-0,17	0,02	0,08	0,04	0,14	0,10	-0,32	-0,04	0,01	-0,11	0,17	-0,13	-0,22	-0,02	0,00	0,04	0,17	0,00	-0,10	-0,05	-0,06	0,08	-0,18	0,36	-0,06	-0,16	0,01	-0,16	0,09	0,06	0,30	-0,12	**0,42**	1,00
**73 (Porphyromonadaceae)**	0,07	0,23	0,18	**0,39**	**0,39**	0,28	-0,16	-0,13	-0,09	0,08	0,20	0,04	0,18	0,04	-0,04	-0,10	-0,13	-0,09	-0,05	0,31	-0,13	0,05	0,15	-0,13	-0,04	-0,11	-0,19	-0,09	-0,08	0,11	-0,03	0,01	0,04	-0,01	-0,09	-0,02	**0,41**	-0,06	0,12	-0,11	-0,18	-0,16	-0,11	-0,10	0,01	-0,16	-0,13	-0,02	-0,04	0,32	**0,39**	0,15	0,06	-0,10	-0,11	-0,14	-0,17	-0,19	0,00	-0,04	0,30	0,24	0,35	-0,11	0,01	-0,07	-0,12	-0,30	-0,02	-0,14	-0,22	-0,12	1,00
**74 (Prevotellaceae)**	0,33	0,26	0,25	0,31	0,30	0,37	-0,01	-0,13	-0,08	-0,01	0,07	0,07	-0,09	-0,15	-0,17	-0,10	-0,10	-0,15	-0,15	0,11	0,06	-0,11	-0,06	-0,19	-0,18	-0,19	-0,08	-0,13	-0,06	-0,16	-0,05	-0,05	0,01	-0,14	-0,09	-0,03	0,01	-0,17	-0,04	-0,04	0,12	-0,12	0,17	0,18	0,01	-0,13	-0,13	0,00	0,00	-0,14	-0,17	-0,18	-0,26	-0,17	-0,08	-0,15	-0,14	0,07	-0,25	0,03	0,16	0,09	0,04	-0,19	-0,01	-0,16	0,29	0,14	-0,04	0,09	0,23	0,22	0,08	1,00
**75 (Ruminococcaceae)**	0,16	0,18	0,17	0,13	0,14	0,14	-0,14	-0,06	-0,32	0,02	0,02	-0,01	-0,02	0,14	0,13	0,17	0,17	0,16	0,09	0,18	0,25	0,17	0,24	0,07	0,18	0,09	0,12	-0,03	-0,23	-0,11	0,20	0,14	-0,05	0,05	0,12	0,15	-0,10	-0,10	-0,35	0,14	-0,29	-0,03	-0,08	0,06	0,04	-0,07	-0,02	-0,16	0,02	0,22	0,16	0,20	0,09	0,05	-0,22	-0,07	-0,13	-0,13	**0,74**	-0,30	0,04	-0,30	-0,37	-0,05	**0,76**	**0,72**	0,26	-0,11	-0,25	**0,70**	**0,67**	0,09	-0,23	0,11	1,00
**76 (S24-7)**	0,36	**0,42**	0,37	0,37	0,33	0,31	-0,03	-0,03	-0,07	0,04	-0,01	0,37	0,12	-0,05	-0,05	-0,09	-0,02	-0,11	-0,12	0,35	0,29	0,23	0,18	-0,20	-0,12	-0,20	-0,17	-0,20	-0,14	0,08	0,27	0,13	0,07	0,12	0,08	0,16	0,19	-0,02	-0,08	0,05	0,02	-0,04	0,09	0,23	0,00	-0,07	-0,03	-0,06	0,10	0,19	0,05	0,23	0,12	0,16	0,00	-0,03	-0,02	0,01	0,30	-0,19	0,28	-0,16	-0,20	0,02	0,42	0,13	**0,85**	0,18	-0,14	**0,48**	**0,61**	0,21	-0,15	**0,48**	**0,51**	1,00
**77 (Spirochaetaceae)**	0,09	-0,04	0,12	0,03	-0,04	-0,01	0,00	0,04	-0,14	0,02	0,02	-0,22	0,17	0,18	0,19	0,23	0,19	0,24	0,12	0,27	0,00	0,05	0,14	0,14	0,21	0,17	0,12	0,10	-0,17	-0,10	0,10	0,04	0,21	0,03	0,25	0,01	-0,10	0,04	-0,22	0,07	-0,15	0,09	-0,08	-0,08	0,07	0,04	0,10	-0,07	0,28	0,13	0,09	-0,01	-0,12	-0,09	-0,01	0,09	0,03	-0,04	0,36	**-0,41**	-0,12	-0,28	-0,22	-0,24	0,19	0,33	-0,17	**-0,38**	**-0,41**	0,02	0,22	0,03	0,07	0,03	**0,42**	-0,06	1,00
**78 (Streptococcaceae)**	0,12	0,02	0,00	-0,10	-0,11	0,03	-0,30	**-0,38**	-0,23	0,05	0,22	-0,25	-0,35	0,03	-0,04	0,18	0,19	0,11	0,03	**-0,38**	0,00	0,00	-0,17	0,10	0,11	0,16	0,26	0,27	0,26	-0,25	0,16	0,25	0,30	**-0,52**	**-0,42**	0,29	-0,33	-0,36	0,14	0,04	-0,11	**-0,42**	-0,17	**0,48**	0,03	**-0,38**	-0,36	0,05	-0,33	-0,30	-0,18	-0,28	-0,31	-0,30	-0,20	**-0,45**	-0,35	0,22	0,01	0,08	-0,29	0,18	0,12	-0,27	0,06	0,12	-0,19	0,12	0,35	0,04	0,01	0,21	0,11	0,25	0,15	-0,10	-0,04	1,00
**79 (Veillonellaceae)**	0,22	0,20	0,18	0,26	0,31	0,34	-0,08	-0,15	-0,04	0,04	0,00	0,16	-0,18	-0,05	-0,04	-0,03	0,02	-0,03	-0,02	0,06	0,24	0,02	0,07	-0,07	-0,11	-0,11	-0,02	-0,07	-0,07	-0,11	0,07	0,09	-0,07	-0,02	-0,06	0,08	0,10	-0,18	-0,17	0,06	0,04	-0,13	0,07	0,21	0,03	-0,16	-0,15	-0,08	-0,03	-0,15	-0,24	0,03	-0,02	0,13	-0,03	-0,12	-0,08	0,15	0,05	-0,20	0,06	-0,19	-0,36	-0,09	0,36	0,16	0,26	0,06	-0,08	**0,48**	**0,61**	0,20	-0,30	**0,68**	**0,45**	**0,57**	0,00	0,04	1,00
**80 (Victivallaceae)**	-0,25	-0,27	-0,31	-0,36	-0,31	-0,24	0,23	0,10	0,07	**-0,39**	-0,34	**-0,59**	**-0,40**	**-0,61**	**-0,63**	**-0,56**	**-0,59**	**-0,54**	**-0,58**	**-0,50**	**-0,44**	**-0,39**	**-0,54**	**-0,47**	**-0,49**	**-0,47**	**-0,39**	**-0,50**	-0,19	-0,27	**-0,42**	-0,21	**-0,39**	-0,13	-0,06	-0,26	**-0,40**	-0,02	0,17	-0,27	0,08	0,06	0,14	-0,11	-0,24	0,12	0,08	0,11	-0,09	-0,18	-0,08	-0,23	-0,22	-0,24	-0,09	0,03	0,03	0,17	-0,29	**0,72**	-0,27	0,22	**0,63**	-0,16	-0,26	-0,20	-0,11	**0,42**	**0,49**	-0,21	-0,28	0,00	0,03	0,16	-0,16	-0,10	-0,21	0,18	-0,10	1,00

Supplementary Table S3. List of bacterial families that significantly correlated to ^{1}H-NMR buckets (|r≥0.37| >, p ≤ 0.05). The central point of ^{1}H-NMR buckets is indicated (ppm) and the potential metabolite that majority contributed to the signal in this region.

Bacterial family	^{1}H-NMR bucket (ppm)	Potential metabolite	r	*p*-value
Bacteroidaceae	4.06	Creatinine	-0.53	0.0049
Bacteroidaceae	0.98	Isoleucine + Valine	-0.43	0.03
Bacteroidaceae	3.06	Creatine + Creatinine	-0.40	0.04
Bacteroidaceae	2.50	Glutamate	-0.40	0.04
Bacteroidaceae	7.30	Phenylalanine	-0.38	0.05
Campylobacteraceae	1.94	Acetate	0.65	0.00028
Campylobacteraceae	1.62	Adipate	0.42	0.03
Campylobacteraceae	0.90	VLDL[a]	0.40	0.04
Campylobacteraceae	2.26	Valine	0.38	0.05
Campylobacteraceae	1.14	3-hydroxybutyrate	0.38	0.05
Campylobacteraceae	3.06	Creatine + Creatinine	0.37	0.05
Campylobacteraceae	2.38	Pyruvate	-0.43	0.02
Clostridiaceae	1.06	Isoleucine	0.40	0.04
Clostridiaceae	3.54	Glucose	0.38	0.05
Coriobacteriaceae	3.54	Glucose	0.55	0.0029
Coriobacteriaceae	3.90	Glucose + AA	0.42	0.03
Coriobacteriaceae	1.14	3-hydroxybutyrate	0.40	0.04
Coriobacteriaceae	5.26	Unsaturated lipids	-0.39	0.05
Desulfovibrionaceae	4.06	Creatinine	0.48	0.01
Desulfovibrionaceae	7.38	Phenylalanine	0.41	0.03
Desulfovibrionaceae	3.66	Ethanol + Isoleucine	0.37	0.05
Enterobacteriaceae	3.86	Glucose + AA	-0.48	0.01
Enterobacteriaceae	3.54	Glucose	-0.40	0.04
Erysipelotrichaceae	3.58	Glucose + AA	0.57	0.0021
Erysipelotrichaceae	2.50	Glutamate	0.49	0.01
Erysipelotrichaceae	2.14	Glutamine	0.49	0.01
Erysipelotrichaceae	7.34	Phenylalanine	0.47	0.01
Erysipelotrichaceae	1.02	Isoleucine + Valine	0.43	0.03
Erysipelotrichaceae	1.06	Isoleucine	0.42	0.03
Erysipelotrichaceae	7.26	Tyrosine	0.40	0.04

Erysipelotrichaceae	1.94	Acetate	0.38	0.05
Lachnospiraceae	3.54	Glucose	0.39	0.04
Lactobacillaceae	2.10	O-acetylglucoprotein	-0.40	0.04
Odoribacteraceae	2.38	Pyruvate	-0.53	0.0042
Odoribacteraceae	3.78	Glucose + AA	-0.50	0.01
Odoribacteraceae	2.50	Glutamate	-0.46	0.02
Odoribacteraceae	4.14	Lactate	-0.44	0.02
Odoribacteraceae	4.06	Creatinine	-0.41	0.04
Odoribacteraceae	3.26	Glucose + TMAO[b]	-0.40	0.04
Odoribacteraceae	2.14	Glutamine	-0.39	0.04
Odoribacteraceae	2.10	O-acetylglucoprotein	-0.39	0.05
Paraprevotellaceae	1.14	3-hydroxybutyrate	0.60	0.00098
Paraprevotellaceae	3.66	Ethanol + Isoleucine	0.54	0.0038
Paraprevotellaceae	1.10	Isoleucine	0.49	0.01
Paraprevotellaceae	3.94	Creatine	0.44	0.02
Porphyromonadaceae	2.10	O-acetylglucoprotein	0.41	0.03
Porphyromonadaceae	7.26	Tyrosine	0.39	0.04
Porphyromonadaceae	1.10	Isoleucine	0.39	0.04
Porphyromonadaceae	7.30	Phenylalanine	0.39	0.05
Streptococcaceae	1.50	Alanine	0.48	0.01
Streptococcaceae	2.26	Valine	-0.52	0.01
Streptococcaceae	0.90	VLDL[a]	-0.45	0.02
Streptococcaceae	1.58	Adipate	-0.42	0.03
Streptococcaceae	3.66	Ethanol + Isoleucine	-0.38	0.05
S24-7	7.38	Phenylalanine	0.42	0.03
Victivallaceae	3.86	Glucose + AA	-0.63	0.00041
Victivallaceae	4.06	Creatinine	-0.59	0.0011
Victivallaceae	3.54	Glucose	-0.54	0.0035
Victivallaceae	3.06	Creatine + Creatinine	-0.50	0.01
Victivallaceae	3.26	Glucose + TMAO[b]	-0.50	0.01
Victivallaceae	2.50	Glutamate	-0.42	0.03
Victivallaceae	3.94	Creatine	-0.40	0.04
Victivallaceae	2.10	O-acetylglucoprotein	-0.40	0.04
Victivallaceae	2.38	Pyruvate	-0.39	0.05

[a]VLDL, very low density lipoprotein; [b]TMAO, trimethylamine-N-oxide.

Supplementary Table S4. Estimated chemical composition of diets, ingredients and additives included.

Lactating diet		Pre-starter diet*	
Net Energy (Kcal/kg)	2450	Net Energy (Kcal/kg)	2480
Crude protein (%)	15.5	Crude protein (%)	16.5
Crude Fat (%)	4.5	Crude Fat (%)	4.8
Crude Fiber (%)	5.7	Crude Fiber (%)	4.1
Lys (%)	1.02	Lys (%)	1.33
Ash (%)	6.70	Ash (%)	5.40
Ca (%)	1.05	Ca (%)	0.55
P (%)	0.60	P (%)	0.61
**Main ingredients:** Barley, Corn, Wheat middlings, Soybean meal, Rapeseed meal, Corn flakes, Lard, Cane molasses, Beet pulp, Sunflower meal, Calcium carbonate, L-Lysine, Sodium bicarbonate, Monocalcium phosphate, Sodium chloride.		**Main ingredients:** Wheat, Barley, Corn Flakes, Oats, Corn, Fish meal, Wheat middlings, Porcine plasma, Soy protein concentrate, Sunflower meal, Soy lecithin, Beet pulp, L-Lysine, L-Valine, Calcium carbonate, Monocalcium phosphate, Sodium Chloride, Choline Chloride.	
**Additives:** Vitamin A (10,000 IU/kg), Vitamin D3 (100 IU/kg); Fe (as $FeCO_3$; 100 mg/kg), Cu (as sulfate pentahydrate; 100 mg/kg); Zn (as ZnO; 100 mg/kg); Se (as sodium selenite, 0.2 mg/kg; and selenomethionine, 0.2 mg/kg); Iodine (as potassium iodide; 0.7 mg/kg); Mn (as MnO_2; 50 mg/kg); 6-Phytase (500 PPU/kg); Butylhydroxytoluene or BHT (0.3 mg/kg); and *Saccharomyces cerevisiae* NCYC Sc47 ($1x10^9$ UFC/kg).		**Additives:** Vitamin A (6,000 IU/kg), Vitamin D3 (1,000 IU/kg); Fe (as $FeCO_3$; 31 mg/kg), Cu (as amino acid chelate; 75 mg/kg); Zn (as ZnO; 50 mg/kg); Se (as sodium selenite, 0.08 mg/kg; and selenite CNCM I-3060, 0.008 mg/kg); Iodine (as potassium iodide; 0.4 mg/kg); Mn (as MnO_2; 25 mg/kg); 6-Phytase (0.63 PPU/kg); Endo-1,4-β-xylanase (3,000 EPU/kg); Butylhydroxytoluene or BHT (0.3 mg/kg); and *Saccharomyces cerevisiae* NCYC Sc47 ($1x10^9$ UFC/kg).	

* Pre-starter diet was also offered as creep-feeding.

Supplementary figures

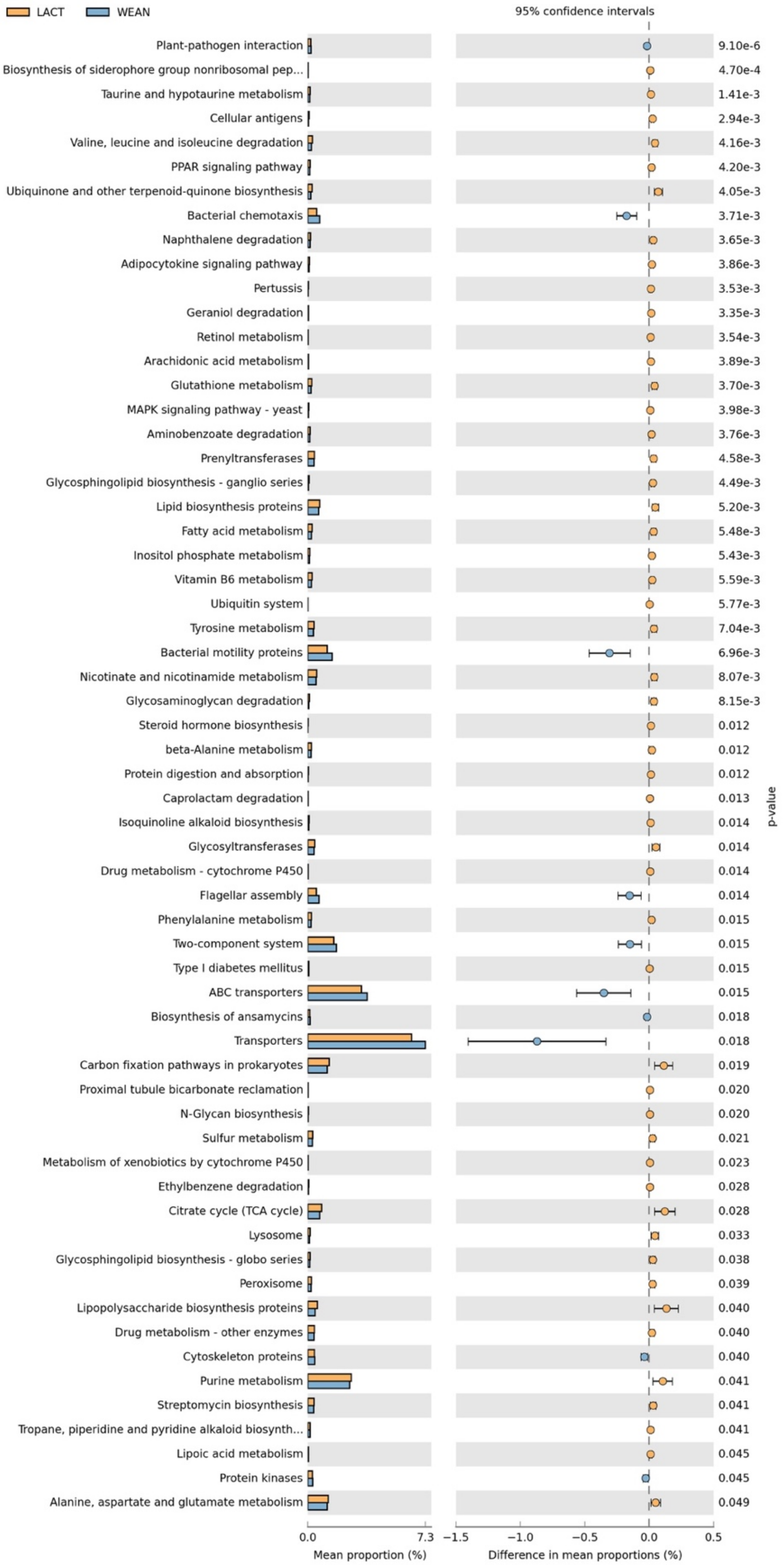

Supplementary Figure S1. Significant differing caecal microbiota pathways between suckling and weaned piglets (KEGG level 3). All sequence reads were used to predict functions against the KEGG database by means of PICRUSt bioinformatics software package. Figure created with the software STAMP.

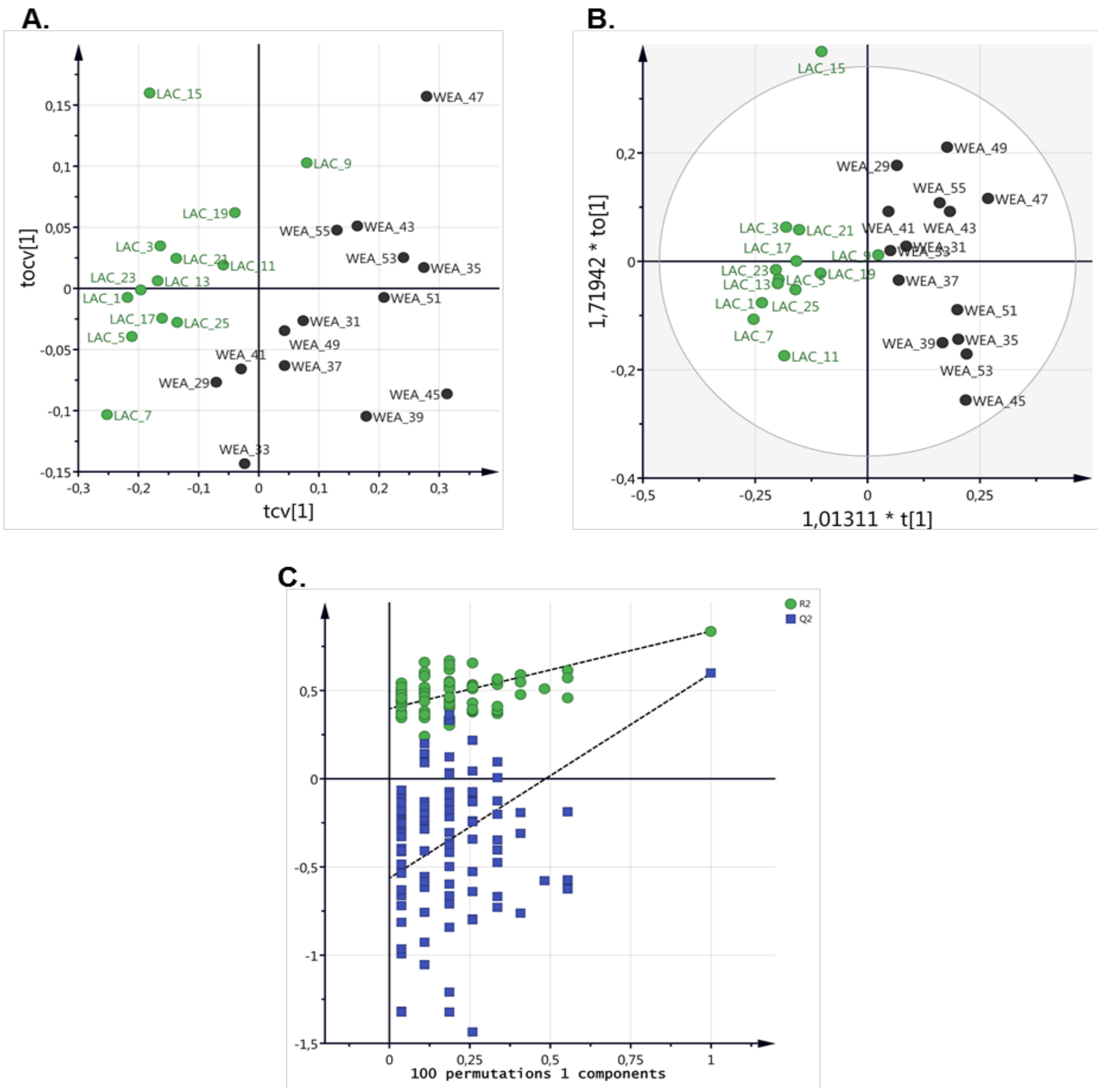

Supplementary Figure S2. Validation of the OPLS-DA model between nursing piglets and after weaning piglets. Cross validation plot (**A**) of the OPLS-DA model. OPLS-DA plot (**B**) derived from ^{1}H-NMR serum spectra of nursing piglets (green) and weaned piglets (black). 100 random permutation test plot (**C**) relative to OPLS-DA model including all samples, where the vertical axis corresponds to R^2 (green circles) and Q^2 (blue squares) values for the model and the horizontal axis corresponds to the correlation coefficient between the original Y and the permuted Y.

The OPLS-DA constructed to discriminate between nursing piglets and after weaning piglets was confirmed by cross validation probe. Comparing both plots, while there was shifting of same spots along the orthogonal axis, the 88.9 % of the spots have the same position respect to the first component, indicating that the OPLS-DA model is devoid of influential observations and it is stable to the inclusion or exclusion of all the different observations. The permutation test plot shows the correlation coefficient between the original y-variable and the permuted y-variable on the x-axis versus the cumulative R^2 and Q^2 on the Y-axis and plots the regression line, the intercept is a measure of the overfitting. The plot of permutation test (100 times) (**Supplementary Figure S1C**) performed for nursing and after weaned piglets shows that the new parameters (R^2=0.40 and Q2= -0.56) were lower than the original values indicating a lack of over-fitting.

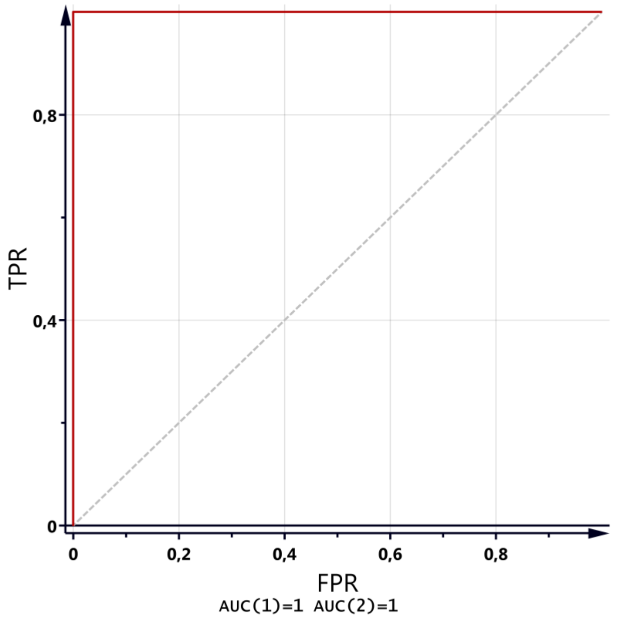

Supplementary Figure S3. Receiver operating characteristic (ROC) plot for the OPLS-DA model between nursing and weaned piglets. The ROC plot displays the TPR for nursing group classification (blue) or for weaned group classification (red) by the constructed model plotted against the corresponding FPR at various threshold settings of the criterion parameter (YPredPS). Both curves have an AUC of the ROC plot of 1.0 indicating high sensitivity and specificity and, in consequence, a high prediction power of the model.

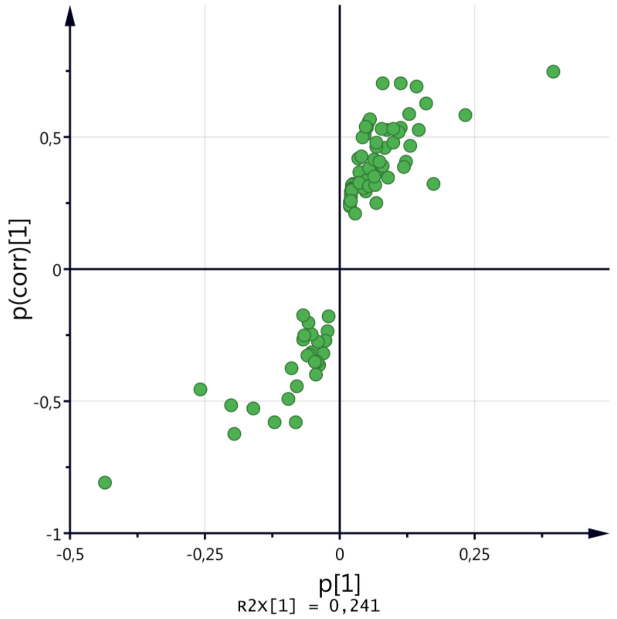

Supplementary Figure S4. S-plot corresponding to OPLS-DA model between nursing and weaned piglets. The covariance value for each variable included is represented on the horizontal axis in the model. The vertical axis represents the correlation values obtained with respect to the dependent variable. The points at both ends of the S-plot curve indicate regions that have a strong discriminant power on the group separation.
